# Persistent and Mobile Chemicals, Including Ultrashort-Chain PFAS, in Groundwater: Distribution, Relevant Factors, and Risk

**DOI:** 10.1021/acs.est.5c13984

**Published:** 2026-02-20

**Authors:** Xiaojing Zhu, Till Meier, Qiuguo Fu, Thorsten Reemtsma

**Affiliations:** † Department of Environmental Analytical Chemistry, 28342 Helmholtz Centre for Environmental Research - UFZ, Permoserstrasse 15, 04318 Leipzig, Germany; ‡ Institute for Analytical Chemistry, University of Leipzig, Linnéstrasse 3, 04103 Leipzig, Germany

**Keywords:** contaminants of emerging concern, drinking water sources, ToxPi scores

## Abstract

Persistent and mobile (PM) chemicals, including ultrashort-chain per- and polyfluoroalkyl substances (PFAS), are continuously discharged into the water cycle, and many of them are poorly removed because of high polarity and recalcitrance. Their occurrence in groundwater, an important drinking water resource, remains underexplored. We investigated 180 PM chemicals in 82 groundwater samples across Saxony, Germany, using freeze-drying and supercritical fluid chromatography-high-resolution mass spectrometry (SFC-HRMS). 163 PM chemicals were determined, including pesticides, pharmaceuticals, PFAS, other industrial chemicals, and transformation products; concentrations of 72 compounds are reported in groundwater for the first time; 57 chemicals exhibited detection frequencies >50%. The median total PM chemical concentration was 23 μg L^–1^, with pesticides (0.77 μg L^–1^) and pharmaceuticals (0.69 μg L^–1^) of similar totals and industrial chemicals being much higher (19 μg L^–1^). Trifluoroacetic acid was the dominant single compound (median of 3.0 μg L^–1^, *n* = 81). Median concentrations of single chemicals correlated with physicochemical properties (water solubility, lipophilicity, etc.). Site-specific factors (nitrate, dissolved organic carbon, depth) were also associated with PM chemical levels. Chemical co-occurrence revealed benzothiazole and *N*,*N*-dimethylformamide as indicators of higher overall PM chemical concentrations. Risk prioritization identified 33 priority chemicals, highlighting chloridazon-desphenyl and *N*-methylpiperidine as potential indicators of higher overall PM chemical risk. These findings clarify PM chemical behavior in groundwater and support refined monitoring strategies.

## Graphical abstract

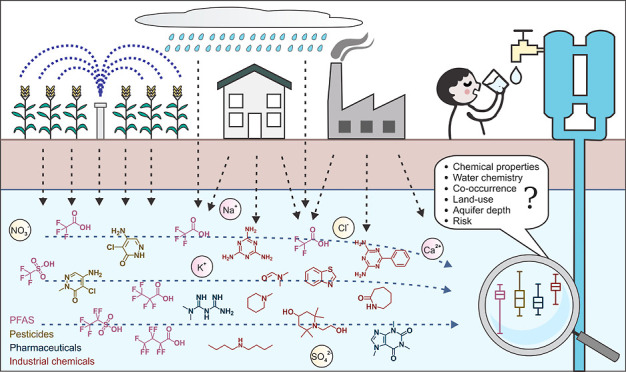

## 1 Introduction

Persistent and mobile chemicals (PMs), including pesticides, pharmaceuticals, industrial compounds along with their transformation products (TPs), and short-chain PFAS (per- and polyfluoroalkyl substances, such as trifluoroacetic acid (TFA), a newly recognized contaminant of concern^1,2^ with increasing occurrence^3–6^ ), have become an increasing public health issue.^7–9^ PMs are prevalent in various environments and potentially toxic, yet their risks were largely underestimated until recent years due to challenges in their detection and analysis.^10^ Water quality monitoring for highly polar compounds by LC-MS (liquid chromatography-mass spectrometry) was long hampered by their limited retention in reversed phase-liquid chromatography.^10^ It requires dedicated approaches for enrichment and chromatography, among them supercritical fluid chromatography (SFC), hydrophilic interaction chromatography, or ion chromatography.^11^ For that reason, PMs remain largely within an “analytical gap”, with quantitative data on the occurrence of PMs in the water cycle being scarce and often limited in the number of compounds included as well as in the geographical scale covered. The full range of chemicals involved is not known yet, and the picture of their distribution and behavior within the water cycle is incomplete.

The high polarity and persistent nature of PMs renders their control in water a difficult task, as it allows them to resist natural degradation and retention processes.^11,12^ Conventional water treatment methods often fail to remove PMs,^13–15^ leading to their ongoing discharge into the environment. Within water cycles, PMs can move along the pathways of water, from field applications and wastewater to surface water and groundwater, and ultimately, drinking water.^12,16–20^

The issue of PMs has taken on even greater urgency with the increasing emphasis on water reuse, a cornerstone of the European Union’s 2020 Circular Economy Action Plan.^21^ As a result, PM contamination now poses a substantial challenge to both water and environmental policy.^22,23^

Groundwater, which provides approximately 70% of the public drinking water supply in Germany, is a crucial resource that faces potential contamination from various sources.^24–28^ In other European countries, surface water is the dominant source of drinking water with up to 80%.^29^ Agricultural activity is known to be a significant source of groundwater contamination with pesticides and their metabolites on a wide scale,^30,31^ while wastewater treatment plant effluents can affect groundwater more locally via surface waters hydraulically connected to aquifers, and, in particular, through bank filtration.^29^ To date, only a limited number of studies have addressed PM contamination in groundwater bodies comprehensively.^20,28^ Much of the previous research has focused on the occurrence of a few specific PMs, without fully exploring the environmental parameters, such as chemical properties, water chemistry, and land use practices, that may govern PM transport to groundwaters.^32^ Consequently, a deeper understanding of PM occurrence in groundwater, as well as the factors that influence their environmental behavior, is needed to guide more effective monitoring and regulatory efforts.

In this study, groundwater samples collected from across Saxony, Germany, (the studied area is about 12,000 km^2^) were analyzed using a method involving freeze-drying and supercritical fluid chromatography-high resolution mass spectrometry (SFC-HRMS), which has been validated across a broad polarity range, including highly polar ones (i.e., log *D* < 0, where log *D* represents the pH-dependent octanol–water partition coefficient).^33,34^ We investigated the occurrence and concentration of a broad set of PMs in groundwater and examined how chemical properties and site properties relate to their occurrence. We further applied a ToxPi-based framework to rank the detected PMs and identify practical indicator candidates for further monitoring, providing insights for future predictive modeling and management of these “gap” chemicals.

## 2 Materials and Methods

### 2.1 PM Selection

Based on the shared suspect lists of PMs published by the Norman Suspect List Exchange,^35^ commercially available PMs were selected for our target list with particular focus on those with low log*D* (pH = 7.4, predicted using ChemAxon Chemicalize), along with their parent compounds and/or TPs. Newly reported PMs that would pose public risk were also included.^19,30,31,36–41^ Compounds found to be unsuitable for analysis by a supercritical fluid chromatography quadrupole time-of-flight mass spectrometry (SFC-QTOF-MS) instrument or with apparent (method) recovery below 20% in representative groundwater matrix spikes (see Text S1.2 and Table S1) were excluded from the final target list. To this end, 180 potential PMs including 33 PFAS (this includes (ultra)­short and novel PFAS), 34 pesticides and TPs, 36 pharmaceuticals and TPs, and 77 industrial chemicals and TPs were selected (see Table S1).

### 2.2 Samples

A number of 82 groundwater samples, covering 25 groundwater bodies (GWBs) across Saxony, Germany, were collected between 2021 and 2024. Detailed information regarding the sampling locations, dates, groundwater body assignments, and known land uses can be found in Table S2. In Saxony, GWBs are delineated according to geological, hydrological, and geohydraulic criteria. Land use information is provided for GWBs by the Saxon State Ministry for Energy, Climate Protection, Environment and Agriculture under the EU Water Framework Directive. The land use classes are qualitative and assigned at GWB scale rather than at individual wells, and we therefore use them only as broad contextual information. Additional data on water chemistry are listed in Table S3. Further details on the groundwater sampling procedure and QA/QC are provided in Text S1.3. Samples were collected as triplicates where possible; in some cases, only single samples were available. Each was stored in a precleaned 50 mL polypropylene (PP) centrifuge tube. They were stored in the dark at 4 °C prior to shipment, transported to our laboratory in insulated boxes with ice packs, adjusted to a final volume of 40 mL, and subsequently frozen at −20 °C in the dark until analysis. Ultrapure water blanks were prepared in the laboratory by filling precleaned 50 mL PP tubes with ultrapure water. These closed tubes were transported and stored together with the groundwater samples throughout sampling, storage, and shipment, and were analyzed with the same workflow to monitor potential contamination from containers, storage, shipment, and handling.

### 2.3 Sample Preparation and Analysis

Samples were freeze-dried, extracted with an azeotropic solvent mixture, evaporated to dryness, and reconstituted to achieve a theoretical 160-fold enrichment, as adapted from our previous studies with some modifications.^33,34^ Briefly, the prefrozen 40 mL aliquots of each sample were freeze-dried (Alpha 1–4 LSCplus, Martin Christ, Osterode am Harz, Germany) for 45 h. The dried samples were then rinsed and extracted three times with 0.5 mL of azeotropic solvent mixture (acetonitrile/water 21/4 v/v) by vortex mixing for 10 s, ultrasonicating for 3 min, and centrifuging at 4,500 rcf for 2 min. The supernatants from the three extractions were combined in a precleaned 2 mL glass vial. This 1.5 mL extract was evaporated to dryness using a TurboVap evaporator (Biotage AB, Uppsala, Sweden) with nitrogen at a flow rate of 0.5 L min^–1^ in a water bath at 40 °C. The resulting residue was reconstituted in 250 μL of solvent mixture (acetonitrile/water 1/1 v/v), vortexed for 10 s, and ultrasonicated briefly for 20 s. The solution was then centrifuged for 30 min at 15,557 rcf at 0 °C to prevent evaporation. Finally, 200 μL of the supernatant was transferred to a new glass vial and stored at 4 °C before analysis by SFC-QTOF-MS within 2 weeks (for instrument setting, reagents, and quality control, see Text S1).

### 2.4 Prioritization of PMs

To prioritize PMs with respect to their health risk, we applied a ToxPi^42,43^ (Toxicological Prioritization Index)-based ranking approach adapted from Hu et al. (2023) and Li et al. (2024).^44,45^ Briefly, ToxPi score was calculated from 17 attributes representing persistence, bioaccumulation, toxicity, and mobility (PBTM, see Text S2 and Table S4).^46^ These scores were then integrated with PM occurrence and concentration data to derive risk indices. PMs were then classified into four priority levels based on risk indices: high (>0.1), medium (0.01–0.1), low (0.001–0.01), and insignificant (<0.001).^44,45^

## 3 Results and Discussion

### 3.1 Overview of the Distribution and Concentrations of PMs

A total of 163 out of 180 PMs (with log *D* (pH = 7.4) values ranging from −6.6 to 4.8, median −0.60, as shown in Figure 1a) were detected in 82 samples, resulting in 13,366 individual concentration data points (163 chemicals × 82 samples). This includes 30 of 33 PFAS, 27 of 34 pesticides and TPs, 33 of 36 pharmaceuticals and TPs, and 73 of 77 industrial chemicals and TPs. Based on our literature review of previously reported PMs in water (summarized in Table S5 and method in Text S3), 14 of the detected PMs were reported for the first time in water, and additional 58 chemicals were reported for the first time with concentrations in groundwater, to the best of our knowledge; together, these 72 substances account for about 44% of all detected PMs in this work. 57 PMs exhibited high detection frequencies >50% (i.e., detected in more than 41 out of 82 samples; Figure 1a). The median summed concentration (*n* = 82) of all detected PMs was 23 μg L^–1^, of which industrial chemicals and their TPs account for 19 μg L^–1^, PFAS for 2.9 μg L^–1^, pesticides and their metabolites for 0.77 μg L^–1^, and pharmaceuticals and their TPs for 0.69 μg L^–1^ (Figure 1b, Table S6).

The individual concentrations and detection frequencies of PFAS and other PMs are shown in Figures S1 and S2, respectively, with complete data available in Table S7. Concentrations previously reported in the literature are summarized in Table S5. Notably, TFA showed the highest median concentration of 3.0 μg L^–1^ based on 81 detections out of 82 samples. Concentrations of a similar order of magnitude have been reported in other water systems, including some drinking water and sources.^1,47–52^ In our data set, TFA contributes the largest share of the summed PFAS concentration, and we therefore report both TFA and the total PFAS concentration separately as descriptive occurrence metrics, without interpreting them as direct measures of health risk. Some of the novel PFAS (such as 2-fluoro-2-(trifluoromethoxy)­acetic acid) were detected at relatively high median concentrations, exceeding 10 ng L^–1^ compared to most PFAS that were around 1 ng L^–1^ or lower, but their detection frequencies were generally low. However, 5H-perfluoropentanoic acid (5-H-PFPeA), showed an unexpectedly higher detection frequency (98%) than perfluoropentanoic acid (PFPeA, 65%), as illustrated in Figure S1 and Table S7. Concentrations of other PMs beyond PFAS ranged from 10 μg L^–1^ to below 0.1 ng L^–1^ (Figure S2).

**Figure 1:**
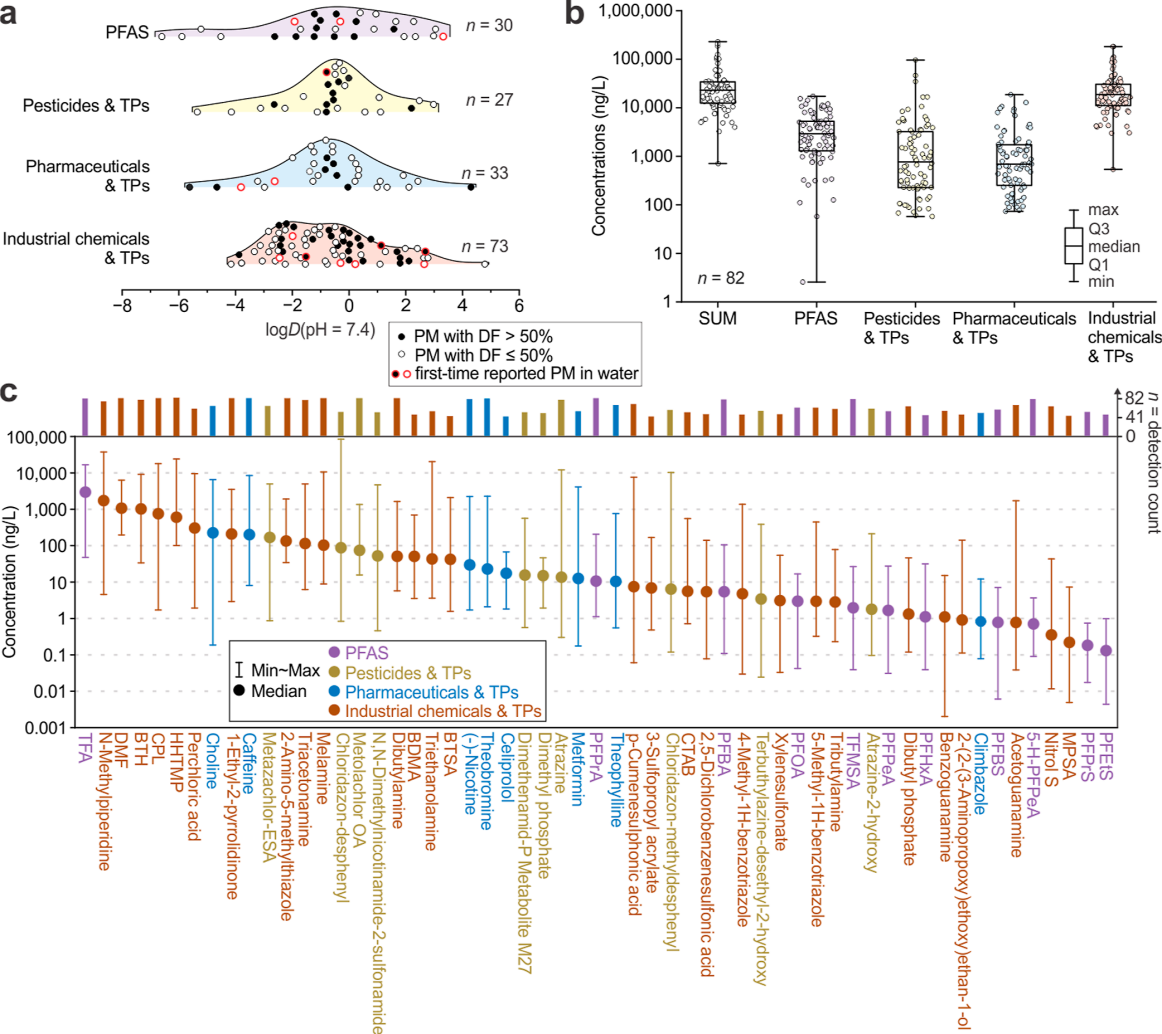
Distribution and concentrations of detected persistent and mobile chemicals (PMs) across four groups: per- and polyfluoroalkyl substances (PFAS), pesticides and transformation products (TPs), pharmaceuticals and TPs, and industrial chemicals and TPs. Panel a: ridgeline plot showing the distribution of Log *D* values (pH = 7.4) of the detected PMs; the *y*-axis represents smoothed relative frequency in arbitrary units. Panel b: summed concentrations of the detected chemicals within each group. Panel c: concentrations of individual chemicals with a detection frequency (DF) exceeding 50%. For the complete and detailed data set, refer to Figures S1 and S2, Tables S6 and S7.

To place pesticide levels in the context of existing regulatory thresholds, we compared their concentrations in the 82 groundwater samples with the drinking-water limit values laid down in Directive (EU) 2020/2184 ^53^ and the German Drinking Water Ordinance of 20 June 2023 ^54^ (0.1 μg L^–1^ for any single pesticide or relevant metabolite, and 0.5 μg L^–1^ for their sum). In our data set, the sum of pesticides and their relevant metabolites exceeded 0.5 μg L^–1^ in 8 of 82 sample, and 20 samples contained at least one pesticide or relevant metabolite above 0.1 μg L^–1^.

Among the 57 chemicals with detection frequencies above 50%, as shown in Figure 1c, TFA exhibits the highest median concentration, followed by three industrial chemicals: *N*-methylpiperidine (which is also an impurity of the agrochemical mepiquat and natural ingredient of roasted coffee),^55,56^ *N*,*N*-dimethylformamide (DMF), and benzothiazole (BTH), each with median concentrations exceeding 1 μg L^–1^. These chemicals play an active role in organic synthesis processes,^57,58^ and their prominence indicates their widespread use, release, transport, and persistence in the environment. Apart from TFA and pentafluoropropionic acid (PFPrA, median concentration of 11 ng L^–1^), most PFAS clustered in the lower concentration range. PFAS-20 and PFAS-4 were both below the drinking water limit values (100 ng L^–1^ and 20 ng L^–1^) also set by Directive (EU) 2020/2184 and German Drinking Water Ordinance of 20 June 2023 (Figure S1).

Some chemicals showed higher detection frequencies and concentrations compared to those of their TPs. For instance, caffeine and its two TPs, theobromine and theophylline (both of them also exist naturally as parent compounds), were widely detected (detection frequencies 100%, 99%, and 82%, respectively), but their concentrations varied by an order of magnitude (median concentrations 200 ng L^–1^, 23 ng L^–1^, and 10 ng L^–1^, respectively). Similarly, atrazine (detection frequency 95%, median concentration 14 ng L^–1^) exhibited levels about ten times higher than its TP atrazine-2-hydroxy (detection frequency 72%, median concentration 1.8 ng L^–1^). In contrast, pesticides such as chloridazon (detection frequency 39%, median concentration 0.80 ng L^–1^), nicosulfuron (detection frequency 9%, median concentration 5.4 ng L^–1^), and metazachlor (detection frequency 9%, median concentration 4.1 ng L^–1^) were detected at significantly lower detection frequencies and concentrations than their respective TPs, chloridazon-desphenyl (detection frequency 63%, median concentration 88 ng L^–1^), *N*,*N*-dimethylnicotinamide-2-sulfonamide (detection frequency 62%, median concentration 52 ng L^–1^), and metazachlor-ESA (detection frequency 79%, median concentration 170 ng L^–1^). This highlights the importance of monitoring both parent chemicals and their TPs in groundwaters. Other parent-TP chemical pairs, such as terbuthylazine and its TP terbuthylazine-desethyl-2-hydroxy, allopurinol and its TP oxypurinol, as well as benzothiazole-2-thiol and its TP benzothiazole-2-sulfonic acid displayed intermediate distribution patterns (see Table S7 and Figure S2 for detailed data). Such differences in parent-TP distribution can be influenced by various factors, including the chemical properties of PMs and their TPs, their biodegradation rates, water chemistry, and land use. These aspects are discussed in detail in the following sections.

### 3.2 Occurrence of PMs and Associated Patterns in Groundwater

#### 3.2.1 Molecular Properties

The physicochemical properties, particularly water solubility and hydrophobicity, are commonly discussed as key descriptors for the distribution and fate of PMs in groundwater.^19,59–61^ Pearson correlation analysis was conducted on the concentrations (median), detection frequencies, and abundances (concentration × detection frequency) of the 163 individual PMs, along with their physicochemical parameters, including water solubility (for very polar or ionizable compounds, the values should be seen as approximate indicators rather than precise solubility limits), lipophilicity (log *D* at pH 7.4), soil–water partitioning (log *K*_oc_), biodegradation half-life, polar surface area (log tPSA), molar refractivity, viscosity, surface tension, and each functional group count, as shown in Figure 2 and Figures S3 and S4.

**Figure 2:**
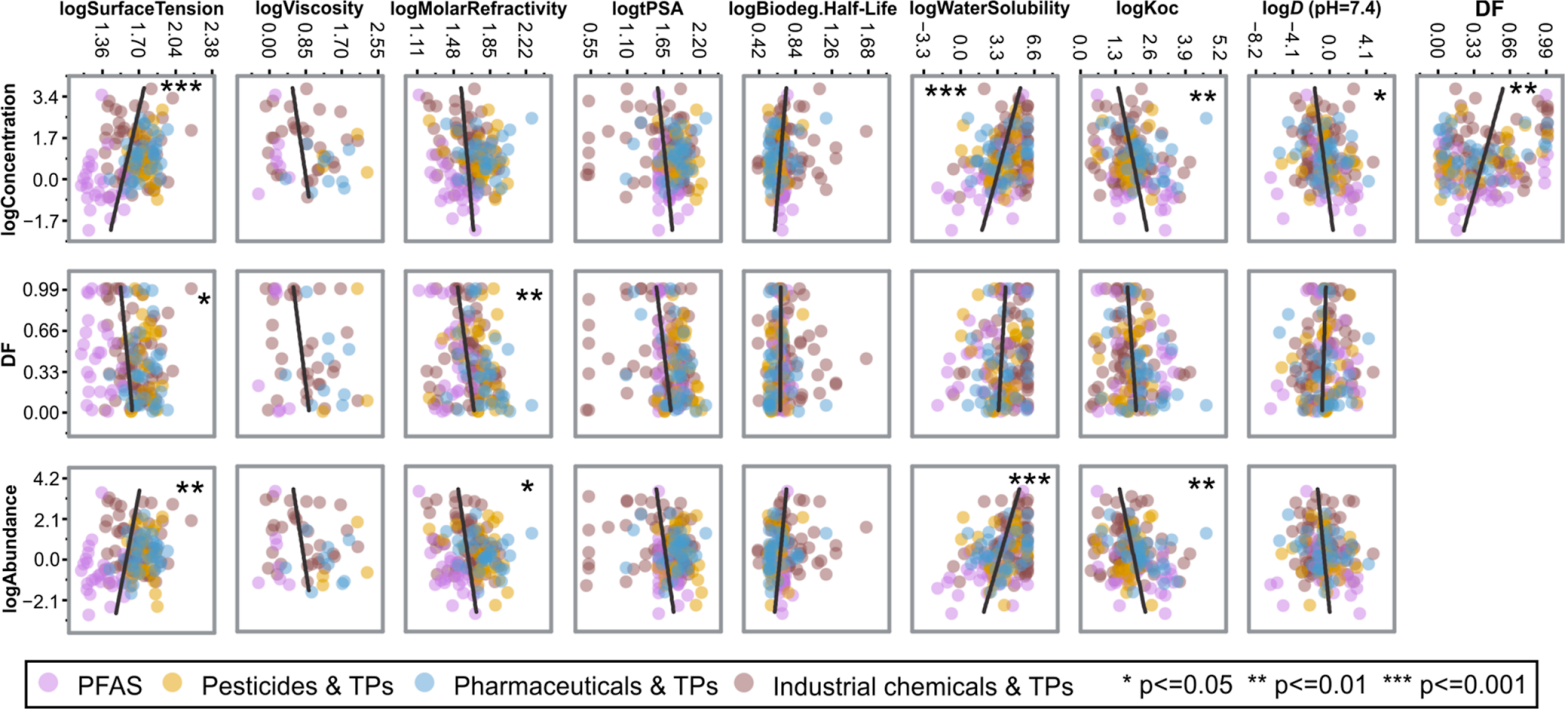
Correlation analysis (Pearson, two-tailed test) between the distribution (median concentrations (ng L^–1^), detection frequency (DF), and abundances) of individual persistent and mobile chemicals (PMs) and their physicochemical properties (*n* = 163). The abundance of each PM was calculated by multiplying the median concentration (*n* = detection count) by its DF. The log *D* (pH = 7.4) was predicted by ChemAxon Chemicalize (https://chemicalize.com/app/calculation), log *K*_oc_ values were obtained from EPI Suite v4.1, and water solubility data was sourced from ECOSAR v2.2; tPSA (topological polar surface area) values were calculated by ChemDraw 20.0, and all the other parameters were extracted from the ComTox Chemicals Dashboard v2.5.2 (https://comptox.epa.gov/dashboard/). The complete correlation results (including the Pearson Corr. (*r*) and *p*-value) between each parameter are depicted as Figure S3. The detailed source data is given in Table S8.

As expected, water solubility exhibited a significant positive correlation with PM concentration and abundance, while log *K*_oc_ and log *D* (pH = 7.4) showed significant negative correlations. This aligns with the expectation that highly soluble chemicals are more likely to remain in the aqueous phase, whereas those with a greater affinity for organic matter tend to adsorb to soil/sediment on their way to groundwater, thereby reducing their concentrations. Molar refractivity (MR), which combines refractive index, molar mass, and density, was also found to have a significant negative correlation with detection frequency and abundance (Figure 2). Although MR is not a direct measure of any single environmental process, it has been used as a proxy for molecular size and polarizability, properties that can influence van der Waals interactions and sorption potential.^62,63^ We acknowledge that MR’s relevance to environmental partitioning is indirect and suggest future work for clearer mechanistic insight. In addition, the surface tension of PMs exhibited a significant positive correlation with median PM concentration and abundance but a negative correlation with detection frequency.

While some physicochemical properties showed significant correlations with median PM concentrations of the respective compounds, their explanatory power is limited due to shallow regression slopes. We therefore use these relationships as pattern-level associations that help to structure the data set and point to possible trends, rather than as proof that any single property controls PM occurrence. The occurrence and concentration of individual PMs in groundwater are mainly governed by where and how much they are used and released (e.g., caffeine^64^), including land-use-related sources. However, systematic data on production volumes, use patterns, emission pathways, and local land use at the well scale are scarce for most of the substances considered here.^65^ Within these constraints, the correlations with physicochemical properties observed in our data set provide additional patterns that may help to interpret differences among compounds.

#### 3.2.2 PM Co-Occurrence

To explore the co-occurrence patterns of PMs in groundwater, correlation analyses were performed for the concentrations of the 57 PMs with detection frequencies exceeding 50% across all sampling sites (Figure S5). Additionally, a correlation network plot was created (Figure S6), illustrating only the very significant correlations (*p* ≤ 0.001, with absolute Pearson coefficients |*r*| ranging from 0.37 to 0.92), and the cluster plot (Figure S7a) highlights the distributional similarities among the chemicals.

Most PMs showed significantly positive correlations to one another, and paired TPs from the same precursors, as well as paired isomers, exhibited highly similar distribution patterns (Figure S7a). These co-occurrence patterns could reflect shared sources, similar environmental behaviors, or both, as co-occurrence patterns can arise from overlapping emission inputs, comparable transport processes, or a combination thereof. Among the 57 PMs with detection frequencies over 50%, two chemicals, BTH (commonly present in rubber materials)^66^ and DMF (a common solvent produced mainly in Germany and widely used throughout the EU in various industries, including pharmaceutical and pesticide manufacturing),^38,67^ were the “central chemicals” showing significantly positive correlations (*p* ≤ 0.001, |*r*| ranging from 0.38 to 0.92) with 32 out of the 57 chemicals (as shown in the correlation matrix, Figure S5, and summarized in Table S9). These two chemicals were also highly correlated with each other (*r* = 0.92, *p* ≤ 0.0001, see Table S9), and the chemicals they correlated with were almost identical and from all the four groups. Following them were four industrial chemicals (melamine (a marker for high levels of urbanization and industrialization),^68^ dibutylamine (detected in groundwater in Denmark),^69^ benzoguanamine (reported in Dutch river bank filtration system and Danish wastewater),^70,71^ and 2-amino-5-methylthiazole), two PFAS (PFPrA and PFBA), and caffeine (recognized as an anthropogenic marker, could indicate rapid groundwater flow and high vulnerability, has been widely detected in groundwater across the EU at levels comparable to the data in this study),^25,26^ each exhibiting significant and positive correlations (*p* ≤ 0.001, |*r*| ranging from 0.37 to 0.85) with at least 20 PMs including BTH and DMF (Figure S5).

A correlation analysis of the total PM concentration and the four grouped concentrations with the top 20 individual PMs (ranked by detection frequencies; see Figure S8) revealed that BTH (*r* = 0.72) and DMF (*r* = 0.66) were among the few compounds showing both strong and significant correlations with the total PM level (*p* ≤ 0.001, *r* > 0.6). Several other high-detection-frequency chemicals, such as melamine, dibutylamine, and theobromine, showed similar correlations. However, TFA, which has the highest median concentration among these 20 chemicals and 99% detection frequency, displayed only a moderate correlation with total PM (*r* = 0.43). In addition, BTH and DMF showed the largest number of significant positive correlations (*p* ≤ 0.001) with other PMs among the 57 chemicals with detection frequencies >50% (as discussed above). Taken together, their strong association with total PM and their central position in the co-occurrence network support their role as practical indicator compounds for elevated PM occurrence in the monitored groundwater bodies (GWBs). Because both compounds can be analyzed well with commonly used chromatographic–mass spectrometric methods, they appear to be attractive candidates for use in tiered monitoring approaches, for example, to obtain an initial picture of PM burdens before more extensive analyses are carried out. BTH is potentially toxic and is poorly removed by conventional drinking water treatment processes,^72^ while DMF has been identified as a precursor to the carcinogen *N*-nitrosodimethylamine during chlorination disinfection.^73^ Presence of these PMs (both chemicals having median concentration exceeding 1 μg/L in our study) in groundwater used for drinking water production further suggests that it requires advanced treatment. Overall, the co-occurrence and network analyses are used here as exploratory tools to visualize shared occurrence patterns among PMs rather than to assign specific sources.

#### 3.2.3 Site Characteristics

To explore whether factors reflected in groundwater chemistry may play a role in shaping PM distribution, we conducted an exploratory correlation analysis between water chemistry parameters and the sum concentrations of the four PM groups at each of the 82 sites (Figure S7b). For pesticides and PFAS, positive correlations were observed with the redox potential and inorganic nitrogen. These parameters are commonly associated with high hydrological connectivity to the surface (e.g., through agricultural input and atmospheric deposition) and may facilitate the input of nitrate and TFA^2,51,74,75^ to shallow groundwater.

Correlations between water characteristics (Figure S9a) and concentrations of individual PMs (detection frequencies >50%, Figure S9b) are summarized in Figures S10 and S11. For example, dissolved organic carbon and pH were negatively associated (*p* ≤ 0.05) with several anionic compounds such as PFAS, nitrol S, dibutyl phosphate, and *p*-cumenesulfonic acid. In contrast, TFMSA exhibited a positive correlation with redox potential and nitrate, suggesting surface connectivity as a driver of its presence. 3-Sulfopropyl acrylate showed higher prevalence in deeper aquifers (detection frequency 58%, median 11 ng/L) than in shallow ones (detection frequency 47%, 7.0 ng/L) and was negatively associated with redox potential and nitrate. Some inorganic ions (e.g., Ca^2+^, Na^+^, Cl^–^, SO_4_^2–^) showed significant (*p* ≤ 0.05) positive correlations with a subset of both anionic (e.g., PFPrS (perfluoropropanesulfonic acid), PFOA (perfluorooctanoic acid), 5-H-PFPeA, nitrol S, dibutyl phosphate, *p*-cumenesulfonic acid, 1-ethyl-2-pyrrolidinone, 2,5-dichlorobenzenesulfonic acid, and xylenesulfonate) and cationic (e.g., *N*-methylpiperidine and DMF) PMs. While the reason behind is unclear, this may reflect cotransport or matrix-driven mobility effects.

Overall, individual pesticides (mainly TPs) showed only limited correlations with water chemistry, indicating that their distribution may be more influenced by land use or transformation history rather than bulk hydrochemistry. Given the complexity and potential interdependencies, a larger data set would be required to draw mechanistic conclusions. In this study, we therefore treat the observed associations between PM levels and groundwater chemistry or depth as pattern-level information that can guide further hypothesis testing.

Groundwater depth affects how strongly PMs are linked to recent inputs from the land surface: shallow screens generally reflect younger, recharge-proximal water, whereas deeper screens tend to intercept older and more buffered groundwater.^76–78^ In this work, sampling depth (upper filter edge) was significantly (*p* ≤ 0.05) negatively correlated with the concentrations of 11 out of the 57 PMs (Figure S5), as well as total PM concentration, total PFAS concentration, and number of detected PMs (Figure S12). For individual chemicals with detection frequencies above 50%, depth was significantly (*p* ≤ 0.05) negatively (*|r* |ranging from 0.29 to 0.45) correlated with the concentrations of six PFAS (TFA, TFMSA, PFPrA, PFBA, PFHxA (perfluorohexanoic acid), and PFOA), two industrial chemicals (melamine and benzoguanamine), two pharmaceuticals (metformin and celiprolol), and one pesticide TP (terbuthylazine-desethyl-2-hydroxy) (Figure S5). Notably, the deepest groundwater investigated in the present work, 102 m below the ground, was the only site where TFA was not detected. These depth-related trends are consistent with the general expectation that shallow groundwater is more exposed to recent inputs from the land surface, while deeper parts of the aquifer system receive more attenuated signals. The observation that TFA was absent only at the deepest site provides a quantitative indication that at least in this regional setting, TFA has penetrated already deeply into groundwaters.

#### 3.2.4 Spatial Distribution and Land Use

To explore the spatial distribution of PMs and potential land use impacts, the concentration data are presented in Figure 3 and Figure S13. These figures display the sum concentrations of different chemical groups at each sampling site, with the corresponding GWB indicated (Tables S2 and S6). Most sites (69) were dominated by industrial chemicals, while 11 were dominated by PFAS.

**Figure 3:**
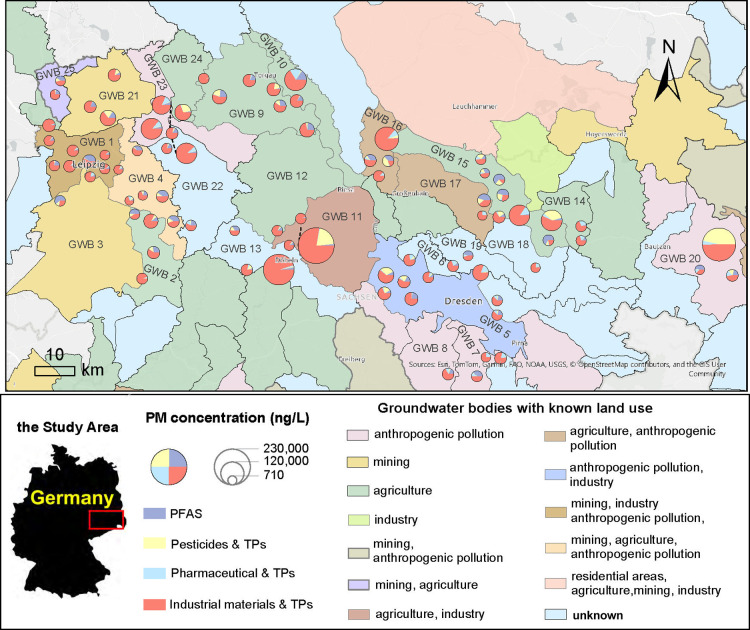
Geographical distribution of the persistent and mobile chemicals as four main groups across the observed groundwater bodies (GWBs) with different known land uses. A column chart version was illustrated as Figure S13 to show the amounts of each PM group clearly, addressing the visual bias in the pie chart when one group dominates. See Table S6 for the detailed data. The data that presents the land use and the boundaries of the GWBs across Saxony, Germany, was extracted from the iDA section of the website of Saxon State Ministry for Energy, Climate Protection, Environment and Agriculture (Sächsisches Staatsministerium für Energie, Klimaschutz, Umwelt and Landwirtschaft, homepage: https://www.umwelt.sachsen.de/umwelt/infosysteme/ida, after clicking on “iDA starten”; access the following link to extract the information: https://www.umwelt.sachsen.de/umwelt/infosysteme/ida/q/2x5ppBQ2O1vxq8h2knlY4y).

Of the 82 sites analyzed, 61 sites exhibited total PM concentrations ranging from 10 to 100 μg L^–1^. At the 7 sites with total PM concentrations >100 μg/L, industrial chemicals dominated (up to 95%), followed by pesticides (up to 47%), pharmaceuticals (up to 17%), and PFAS (up to 16%). In contrast, at the 14 sites with low total concentration (<10 μg/L), industrial chemicals still dominated (up to 87%), followed by PFAS (up to 48%), pharmaceuticals (up to 23%), and pesticides (up to 14%), and these sites are linked to multiple land uses. In the 11 sites dominated by PFAS, total PM levels typically ranged from 10 to 100 μg L^–1^, and the primary land use was agricultural (information on the known land use of each GWB was obtained from the Saxon State Ministry for Energy, Climate Protection, Environment, and Agriculture, Table S2). Overall, the concentration patterns observed across sites show that PMs occur over a wide concentration range, involving different chemical groups and covering the GWBs of various land use categories (see Section 2.2 for details). Within this framework, elevated total PM levels were observed in GWBs assigned to several different land use categories rather than being restricted to a single one. The land use information is used here as broad contextual guidance rather than as a quantitative predictor. More detailed source-receptor analyses (for example, relating concentrations to distances from specific industrial or agricultural activities) would require additional spatial data and are a logical next step for future work.

To investigate spatial distribution patterns, we performed a cluster analysis of the concentration of all 163 detected PMs, by averaging data from multiple sampling sites within each of the 25 GWBs. The resulting circular clustered heatmap (Figure 4) provides an overview of PM patterns across the 25 GWBs and highlights groups of chemicals that occur preferentially within each of the two main GWB clusters. In the purple/inner cluster, certain chemicals were exclusively detected, including five industrial chemicals (aspartame, 3-(allyloxy)-2-hydroxypropanesulfonate, naphthalene-1-sulfonic acid, 3,4-diaminobenzoic acid, and 2-amino-4,5-dichlorobenzenesulfonic acid), two PFAS (PFPediA, i.e., hexafluoroglutaric acid, and difluoropropanedioic acid), two pharmaceuticals (propranolol glucuronide and allopurinol), and one pesticide (mesotrione). In contrast, some chemicals were found only in the green/outer cluster, such as two industrial chemicals (tripropylamine and ethylpyridinium), two PFAS (3-chlorotetrafluoropropionic acid, and perfluoro-3-methoxypropanoic acid), two pharmaceuticals (erythromycin and amisulpride), and five pesticides (metazachlor, nicosulfuron, mecoprop, mecoprop-P, and 4-(methylsulfonyl)-2-nitrobenzoic acid). Several of these, like aspartame and PFPediA, were found in over half of the GWBs in their respective cluster, suggesting potential as region-specific or activity-related indicators.

**Figure 4:**
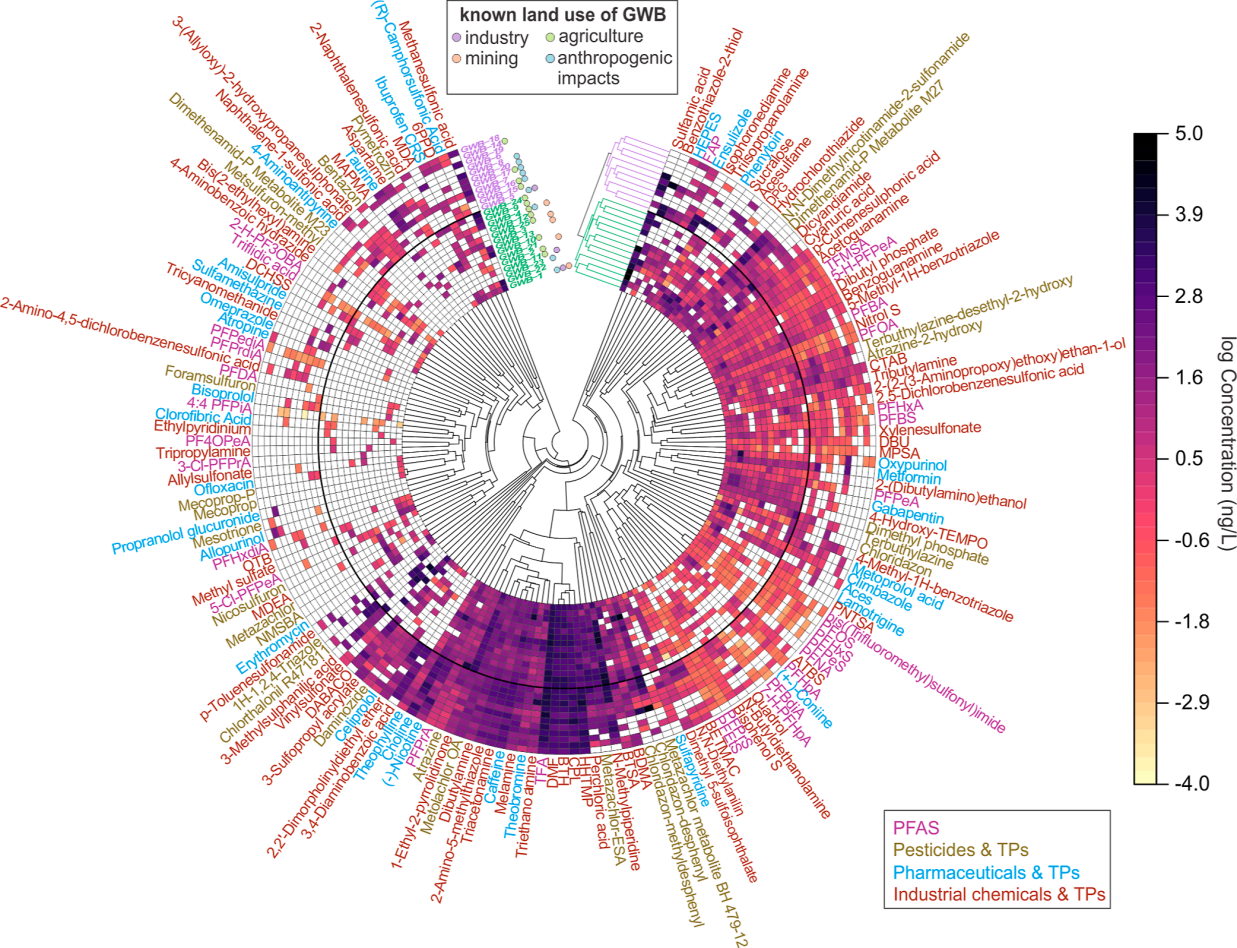
Circular cluster analysis of the occurrence of 163 detected persistent and mobile chemicals (PMs) in 25 groundwater bodies (GWBs) in Saxony. The circular layout displays the concentration matrix between individual PMs and GWBs; each colored segment represents the concentration of one chemical in one GWB, with color intensity increasing from low to high concentration (white denotes nondetections). Hierarchical clustering was applied to both GWBs and chemicals, and the associated dendrograms and colored bands separate the 25 GWBs into two main clusters (outer/purple and inner/green) and group chemicals with similar spatial occurrence patterns across the GWBs. The detailed data can be found in Table S10.

The occurrence of similar chemical profiles among grouped GWBs suggests that they may be subject to comparable land use conditions. This may help identify potential regional influences, particularly in GWBs where direct information about contamination sources is lacking or where transboundary aquifers complicate local source attribution. For example, GWB 22 clustered closely with GWB 23, implying that both are likely influenced by similar pollution pressures, possibly due to a hydraulic connection or shared upstream sources. While these spatial patterns provide insights into potential land-use-related pressures, they do not constitute direct evidence of specific pollution sources, as subsurface transport and hydrogeological conditions can vary greatly. Monitoring data are assigned to GWBs based on the existing network and spatial delineation, but source–pathway relationships may differ considerably due to local hydrogeological complexity. These relationships are based on the present regional data set and should be regarded as exploratory; they may differ under other hydrogeological and land use settings. More intensive sampling and monitoring would be helpful to test relationships between land-use and PM concentrations in a statistically robust way.

### 3.3 Potential Risk of PMs

To prioritize PMs with respect to their health relevance, we used the ToxPi-based approach described in Section 2.4 (see also Table S11). The exposure and risk indices are normalized to the range of concentrations and detection frequencies across the 163 substances, so they provide a screening-level prioritization within this monitoring data set. The use of maximum concentrations follows the published framework and is intended as a conservative indicator of upper-bound levels within the data set; it does not imply that these maxima are typical for the entire study area. This ranking, in the present work, is used as an exploratory tool to structure the large set of detected PMs and to identify candidates for more detailed assessment rather than as a definitive risk evaluation. This yielded risk indices and priority levels for all 163 PMs (summarized in Table S12 and visualized in Figure S14). 33 PMs were prioritized with a risk index ≥ 0.001 and a detection frequency above 50% (Figure 5a, full PM list in Figure S15), including TFA as the only prioritized PFAS. Also prioritized were 7 pesticides, 6 pharmaceuticals, and 19 industrial chemicals. The top-ranked compound was a pesticide TP chloridazon-desphenyl (high risk index), followed by three industrial chemicals, including *N*-methylpiperidine (high risk index), 4-hydroxy-1-(2-hydroxyethyl)-2,2,6,6-tetramethylpiperidine (HHTMP, medium risk index), and ε-caprolactam (medium risk index), all detected in 100% of the GWBs at concentrations of 100 ng/L to 10 μg/L. Inspection of the PBTM profiles in Figure 5a shows that the higher risk indices of these top-ranked substances are mainly associated with comparatively high toxicity and mobility segments, together with their frequent detection and elevated concentrations. For chloridazon-desphenyl and *N*-methylpiperidine, the toxicity segment is largely driven by predicted repeated-dose toxicity and developmental toxicity, with additional contributions from some qualitative human-health end points such as carcinogenicity or mutagenicity (Table S12). HHTMP and ε-caprolactam display a similar pattern, although with somewhat smaller toxicity contributions.

**Figure 5:**
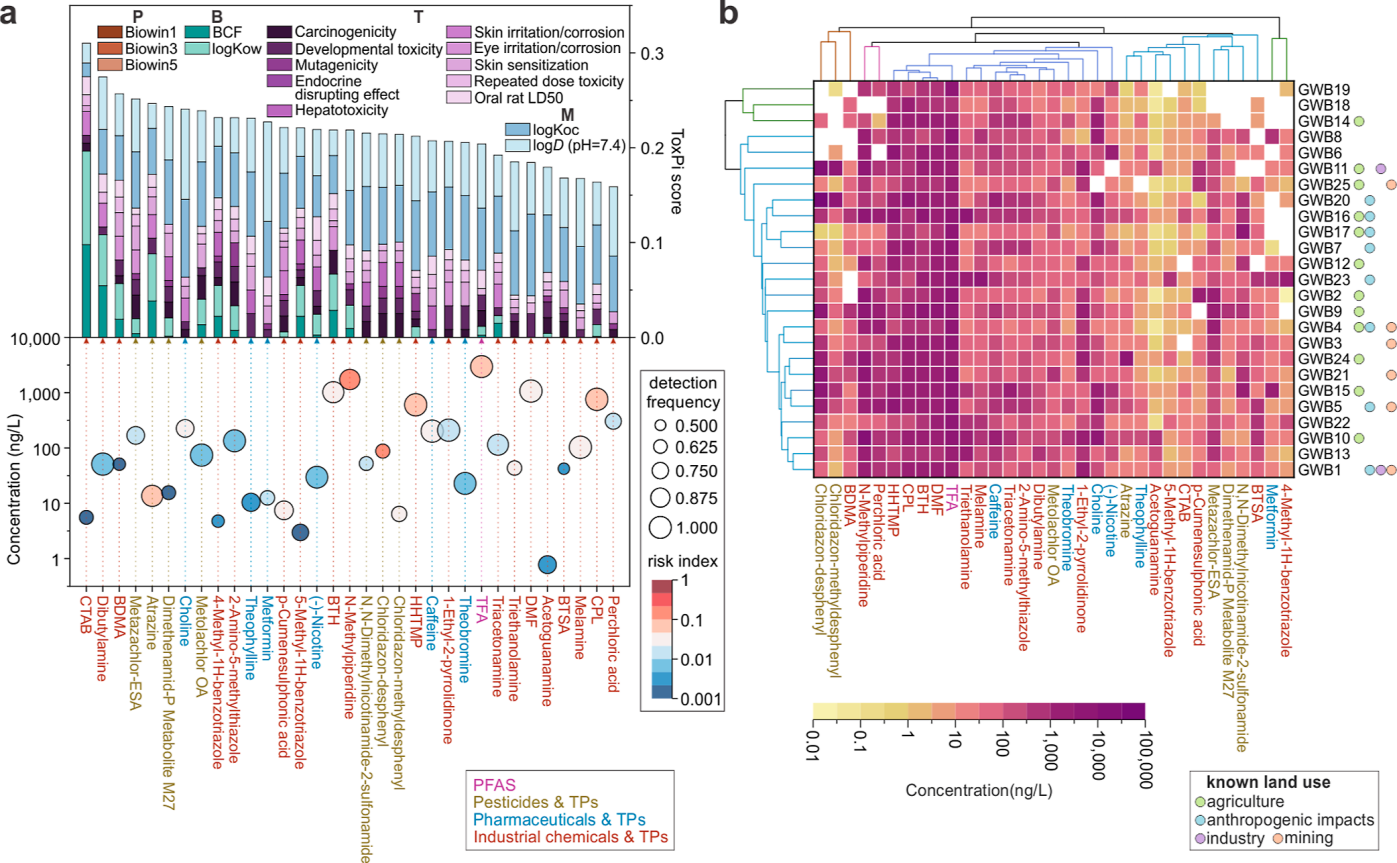
(a) ToxPi scores of the 33 prioritized persistent and mobile (PM) chemicals with risk indices greater than 0.001 and detection frequencies exceeding 50%. The concentrations used correspond to the median values across all detected samples. P: persistence, B: bioaccumulation, T: toxicity, M: mobility. (b) Cluster analysis showing the distribution of these 33 prioritized PMs in the monitored groundwater bodies (GWBs). For GWBs with multiple sampling sites, mean concentrations of individual PMs were utilized. Refer to Tables S10 and S12 for detailed data.

Based on the concentration data set of the prioritized PMs across the monitored GWBs, we performed two complementary analyses: (i) a cluster analysis to group GWBs by similarity in PM occurrence (Figure 5b), and (ii) a risk score calculation, where the concentration of each prioritized compound was multiplied by its individual risk index to estimate cumulative risk per GWB (Figure S16). Among the 25 GWBs, two (GWB 11 and GWB 20) had the highest risk scores, primarily due to chloridazon-desphenyl. As this compound is a known pesticide TP, its dominance suggests possible agricultural influence on these GWBs. Six GWBs (GWBs 9, 10, 15, 16, 21, and 24) showed medium risk scores, driven by contributions from both *N*-methylpiperidine and chloridazon-desphenyl, indicating a potential combined influence from industrial and agricultural sources. The remaining GWBs, which had lower scores, were primarily dominated by mixtures of TFA, *N*-methylpiperidine, HHTMP, and melamine, suggesting inputs from multiple sources. These risk-based groupings are also reflected in the clustering of the prioritized chemicals shown in Figure 5b, where compounds associated with high (e.g., chloridazon-desphenyl), medium (e.g., *N*-methylpiperidine), and lower risk scores (e.g., TFA, HHTMP, melamine) form distinct clusters based on their distribution patterns across GWBs. Note that this framework, while a valuable tool for integrating hazard-relevant properties, is not designed to identify pollution sources. Therefore, we interpret source associations as hypothesis-driven and exploratory rather than definitive.

While the ToxPi-based prioritization is useful for screening, certain results warrant careful interpretation. Some substances with high scores are naturally occurring. Their hazard classifications are based on predictive databases and may not reflect regulatory concern. These limitations will be considered in future refinements of the approach.

## 4 Implications

This work reveals that a wide variety of PMs is occurring widely in groundwater in Saxony, Germany, an important drinking water resource, posing a challenge for water quality management. While the data are specific to this region, the occurrence patterns and the set of frequently detected PMs may also be informative for other regions with similar hydrogeological and emission conditions. For pesticides and their relevant TPs, the sum limit value of 0.5 μg L^–1^ was exceeded in 8 of 82 samples, and several individual substances exceeded the 0.1 μg L^–1^ limit at 20 sites (Section 3.1).

The widespread presence of PMs beyond pesticides and their metabolites, including the ultrashort-chain PFAS TFA, pharmaceuticals, and various industrial compounds and their TPs, shows that groundwater can contain complex mixtures of PMs. For many of these PMs, health-based drinking water guideline values are not available. The present data identify substances with frequent occurrence that may merit further toxicological evaluation. BTH and DMF emerged as promising indicator candidates for an elevated total PM level in the monitored GWB. The frequent detection of TPs suggests that they deserve more attention in future monitoring and risk evaluation.^79^ This study does not attempt a full health risk assessment for individual sampling points; instead, it focuses on occurrence patterns and screening-level prioritization.

The distribution of PMs reflects a complex interplay of factors. Key molecular properties include water solubility, *K*_oc_, and log *D*, while certain land use patterns are also linked to PM occurrence and distribution. However, these interactions are not yet fully understood. Land use is a broad contextual factor that may relate to groundwater PM levels differently depending on recharge pathways (e.g., precipitation, surface water, and irrigation), subsurface transport, and management practices. Further research, combining PM monitoring with hydrogeological characterization and process-based studies, is needed to clarify these relationships.

The prioritization framework, based on persistence, bioaccumulation, toxicity, mobility, and distribution, identified chemicals such as *N*-methylpiperidine and chloridazon-desphenyl as potential indicators of elevated PM risk. While useful for screening, it is not intended to pinpoint sources.

The following activities would be instrumental to improve source understanding and risk assessment for PMs. (i) Monitoring programs could combine broad PM screening with a small set of indicator substances, applied in denser spatial and depth-resolved sampling networks. (ii) Better information on production volumes, use patterns, point sources, and hydrogeological setting would help to relate observed groundwater patterns to emission sources. (iii) Refinement of prioritization tools such as the present ToxPi-based index will also require more experimental or curated toxicity data and transparent links to health-based guidance values. The data set presented here offers a starting point for such developments in Saxony and may be informative for regions with comparable conditions.

## Supplementary Material





## Notes

Additional tables detailing the chemical properties and analytical parameters, groundwater sampling location and properties, quantified results of the PMs in the groundwaters, and data used for ToxPi score and risk index calculation as well as the results (XLSX)
Additional details related to ToxPi and risk index calculation, and all the supplementary figures displaying the results of PMs in groundwaters (PDF)
