# Supplementary material for: Persistent and Mobile Chemicals, Including Ultrashort-Chain PFAS, in Groundwater: Distribution, Relevant Factors, and Risk
Source: Environ Sci Technol. 2026 Feb 20;60(8):6649–61. doi: 10.1021/acs.est.5c13984 (PMC12961943; doi:10.1021/acs.est.5c13984)
Supplement: Supplementary file 2 [file es5c13984_si_002.pdf]

Supporting Information of

Persistent and mobile chemicals, including ultra-short-chain PFAS, in groundwater: distribution, relevant factors, and risk

*Xiaojing Zhu [a], Till Meier [a], Qiuguo Fu [a], Thorsten Reemtsma [a,b] **

[a] Department of Environmental Analytical Chemistry, Helmholtz Centre for Environmental Research - UFZ, Permoserstrasse 15, 04318, Leipzig, Germany

[b] Institute for Analytical Chemistry, University of Leipzig, Linnéstrasse 3, 04103 Leipzig, Germany

*Corresponding author:
Thorsten Reemtsma, Email: thorsten.reemtsma@ufz.de

List of content (22 pages)

Text S1
Text S2
Text S3
Table S4
Fig. S1
Fig. S2
Fig. S3
Fig. S4
Fig. S5
Fig. S6
Fig. S7
Fig. S8
Fig. S9
Fig. S10
Fig. S11
Fig. S12
Fig. S13
Fig. S14
Fig. S15
Fig. S16

Text S1

S1.1 Chemicals and reagents

Ultrapure water (UHPLC–MS grade) from Thermo Scientific™, acetonitrile (LC–MS Ultra grade) from Honeywell CHROMASOLV™, and methanol (UPLC/MS - CC/SFC grade) from Biosolve™ were used for sample preparation. Ammonium formate and formic acid were both purchased from Biosolve™. Information of all the reference standards can be found in Table S1.

S1.2 Instrument analysis

The enriched samples were analyzed using SFC–QTOF–MS, which consisted of an ACQUITY UPC^2 system coupled with a XevoG3 QTOF (Waters, Eschborn, Germany). The inlet and MS methods were adapted and optimized based on our previous work (ref 32 and 34 of the main text). For chemical analyses in positive and negative electrospray ionization (ESI) modes, separations were carried out on a BEH column (3.0 mm × 100 mm, 1.7 µm) and a Torus Diol column (3.0 mm × 100 mm, 1.7 µm), respectively, both from Waters, Eschborn, Germany, maintained at 55 °C with an injection volume of 5 µL. Chromatographic separation occurred at a constant flow rate of 1.3 mL min^{-1} using supercritical CO_2 as eluent A and methanol:water 95:5 with 10 mM ammonium formate as eluent B (modifier) for the mobile phase. The gradient was programmed over a 19-minute run as follows: 0 min, 1% eluent B; 0.2 min, 1% eluent B; 8.5 min, 25% eluent B; 12 min, 50% eluent B; 17 min, 50% eluent B; 17.1 min, 1% eluent B; 19 min, 1% eluent B. A backpressure of 2200 psi was maintained to ensure supercritical conditions. Post-column, a make-up flow of methanol containing 0.1% formic acid at 0.15 mL min^{-1} was introduced to direct the effluent into the ESI-MS interface.

The MS settings were configured with a capillary voltage of 0.5 kV for negative ion mode and 0.8 kV for positive ion mode. The sampling cone voltage was set at 10 V for negative ion mode and 30 V for positive ion mode, with the source offset matching these values (10 V and 30 V, respectively). The source temperature was maintained at 120°C, while the desolvation temperature was set at 600°C, and the cone gas flow was 1,000 L h^{-1}. MS^E acquisition was used to gather two data sets simultaneously: a low-collision-energy scan (10 eV) to capture parent ion information and an elevated-collision-energy scan (from 15 to 40 eV) to obtain fragment ions. The data were acquired in centroid mode with a scan time of 0.1 seconds, covering a mass range of m/z 50–1,200 at a resolution of 22,000. A lock-spray containing leucine enkephalin at a concentration of 100 pg µL^{-1} was continuously infused throughout the measurement for calibration.

Data processing was carried out using the TargetLynx module in Waters MassLynx v4.2 software, applying peak integration methods, containing the retention times and the quantification traces, created by individual chemical reference standards. Quantification was performed using 9-point calibration curves prepared in a solvent mixture of acetonitrile and ultrapure water (1/1, v/v). Concentrations in groundwater were calculated from the calibration curves, taking into account the method enrichment factor and the apparent recovery of each persistent and mobile chemical (PM, for detailed description of mathematical description of apparent recovery, see our previous work (ref 32 of the main text)). Apparent recovery was determined from matrix spike experiments using a representative groundwater (GW1), selected for its minimal contamination and relevance to drinking water production. Mixtures of all PMs were spiked into GW1 at known concentrations and processed through the complete workflow (freeze-drying enrichment and SFC–QTOF–MS analysis). For each analyte, the mean apparent recovery and its (relative) standard deviation (SD and RSD) derived

from 6 replicates are reported in Table S1. These values were used both to exclude compounds with apparent recovery below 20% from the final target list and to characterise the uncertainty associated with recovery-corrected concentrations of the retained analytes.

S1.3 Groundwater sampling and quality assurance/quality control (QA/QC)

Groundwater sampling was carried out by the Saxon monitoring authorities LfULG (Saxon State Office for Environment, Agriculture and Geology) and BfUL (Staatliche Betriebsgesellschaft für Umwelt und Landwirtschaft, Radebeul, Germany) following the standard protocol of the Saxon groundwater monitoring network. In brief, the monitoring well was opened and a pump was installed above the filter section. Groundwater from the well and the immediate vicinity of the filter section was first pumped and discarded to avoid sampling water influenced by the well itself. During pumping, water quality parameters (e.g., pH, dissolved oxygen, turbidity) were continuously monitored. Once these parameters had stabilized, indicating a consistent composition of the pumped water, samples were taken and the corresponding 50 mL polypropylene tubes were filled. The entire pumping and sampling process, including flow rates, was documented by the responsible authority.

To prevent contamination, glass vials and inserts were heated at 500 °C for 4.5 hours in a muffle furnace, exclusively used for cleaning glass or metal labware, prior to use. All glassware used for solvent transfers and eluent/make-up flow preparation, such as beakers, measuring cylinders, and bottles, were thoroughly cleaned and reserved solely for this purpose.

Several types of blanks were used to monitor potential contamination and carry-over: (i) transport/handling blanks, consisting of ultrapure water filled into pre-cleaned 50 mL polypropylene tubes in the laboratory, closed and transported/stored together with the groundwater samples (see Section 2.2); (ii) procedural blanks, in which ultrapure water was processed through the complete workflow (freeze-drying enrichment and SFC–QTOF–MS analysis); and (iii) solvent and instrument blanks, injected between batches in the SFC–QTOF–MS sequence to check for background and carry-over. Target PMs were not systematically detected above reporting limits in any blank type; the blanks were therefore used qualitatively to confirm the absence of significant contamination, and no numerical blank subtraction was applied.

S1.4 Statistical analysis

Details on the statistical analyses and the corresponding sample sizes (*n*) for each section are presented in the figure legends. The analyses were carried out using Origin 2024 and Microsoft Excel 2016.

When interpreting absolute concentrations, especially for compounds with low apparent recoveries and higher RSDs (Table S1), the additional uncertainty introduced by recovery correction should be taken into account.

Text S2 Calculation of ToxPi (Toxicological Prioritization Index) score and risk index

The prioritization framework for ToxPi scores and risk indices used in this study was adapted from Hu et al. (2023) and Li et al. (2024) (ref 44 and 45 of the main text). We considered 17 attributes, classified into four categories: persistence (P), bioaccumulation (B), toxicity (T), and mobility (M)

(details in Table S10).

Persistence (P) included three attributes: Biowin 1 (probability of rapid biodegradation based on a linear probability model), Biowin 3 (estimated time for "complete" ultimate biodegradation under typical aerobic aquatic conditions using an expert survey ultimate biodegradation model), and Biowin 5 (biodegradability based on a linear model similar to Biowin 1, reflecting results from the Japanese Ministry of International Trade and Industry ready biodegradation test). These were obtained using BIOWIN v4.11 from the EPI (Estimation Programs Interface) Suite v4.1 (U.S. EPA, https://www.epa.gov/tsca-screening-tools/epi-suitetm-estimation-program-interface).

Bioaccumulation (B) was assessed using bioconcentration factor (BCF) and logK_{ow} (octanol-water partition coefficient), sourced from BCFAF v3.02 and KWOWIN v1.69, respectively, within the EPI Suite v4.1.

Toxicity (T) comprised attributes related to human health effects, including ten attributes: carcinogenicity, developmental toxicity, mutagenicity, endocrine-disrupting effects, hepatotoxicity, skin irritation/corrosion, eye irritation/corrosion, skin sensitization, repeated dose toxicity, and oral rat LD50. These were obtained from VEGA v1.2.3 (Laboratory of Environmental Chemistry and Toxicology, Istituto di Ricerche Farmacologiche Mario Negri IRCCS, https://www.vegahub.eu/), T.E.S.T. (Toxicity Estimation Software Tool) v5.1.2.0 (U.S. EPA, https://www.epa.gov/chemical-research/toxicity-estimation-software-tool-test), and QSAR ((Quantitative Structure–Activity Relationship models) Toolbox v4.6 (https://qsartoolbox.org/). Toxicity attributes not available from qualitative models were assigned a value of zero. For attributes such as carcinogenicity, developmental toxicity, mutagenicity, endocrine-disrupting effects, and skin sensitization, average qualitative endpoint values (0 for negative/inactive, 1 for positive/active) were used from different models/software. Repeated dose toxicity was represented by the no-observed-adverse-effect level (NOAEL), the highest exposure level without biologically significant adverse effects in the exposed population compared to controls.

Mobility (M) attributes included logK_{oc} (organic carbon-water partition coefficient) and log$D$ (pH=7.4) (pH-dependent lipophilicity). logK_{oc} was sourced from KOCWIN v2.1.1 of EPI Suite v4.1, and log$D$ was obtained from the ChemAxon Chemicalize platform (https://chemicalize.com/app/calculation).

To normalize data distribution, some attributes underwent log transformation (Table S10 provides scaling details). ToxPi scores of the chemicals were then calculated using the ToxPi software (https://toxpi.org/), allowing hazard ranking based on the selected attributes.

For calculating the risk index of each chemical, its detection frequency (DF), magnitude (represents the relative intensity of each chemical), exposure and its normalized version ($exposure_{normalized}$), and ToxPi Score were used based on the equations below:

$$Magnitude = \frac{C_i - C_{min}}{C_{max} - C_{min}} \quad (1)$$

$$Exposure = \mathrm{DF} \times Magnitude \quad (2)$$

$$Exposure_{\mathrm{normalized}} = \frac{Exposure_i - Exposure_{min}}{Exposure_{max} - Exposure_{min}} \quad (3)$$

$$\text{Risk index} = \text{ToxPi Score} \times Exposure_{\mathrm{normalized}} \quad (4)$$

where C_i represents the maximum concentration of chemical i, C_{max} is the highest maximum

concentration among all chemicals, C_{min} is the lowest maximum concentration among all chemicals, *Magnitude* denotes the concentration magnitude of a chemical, $Exposure_i$ is the exposure value of chemical *i*, while $Exposure_{min}$ and $Exposure_{max}$ represent the minimum and maximum exposure values among all chemicals, respectively.

These equations correspond to three conceptual components: (i) *Magnitude* expresses how high the maximum concentration of each chemical (C_i) is relative to the other PMs in this study, using min-max normalization across all compounds; (ii) *Exposure* combines this concentration magnitude with detection frequency (DF), so that substances that are both widespread and elevated obtain higher exposure indices than substances that are rare or only show isolated high values; and (iii) the risk index combines the normalized *Exposure* ($Exposure_{normalized}$) with the ToxPi score to obtain a dimensionless ranking of PMs based on both hazard-related attributes and occurrence. By construction, the *Magnitude*, *Exposure* and risk indices are relative to the set of PMs included in this monitoring (n = 163), and are therefore intended for prioritization within this dataset rather than for direct comparison with other studies that use different chemical panels.

To illustrate the calculation, we provide an example for one representative compounds, benzothiazole (BTH):

For BTH, the maximum concentration observed in our dataset is C_i = 9,154 ng L^{-1} and the detection frequency is DF = 95%. Given C_{max} = 155,324 ng L^{-1} and C_{min} = 0.001 ng L^{-1} across all 163 PMs, the Magnitude of BTH is:

$Magnitude_{\mathrm{BTH}} = (C_i - C_{min}) / (C_{max} - C_{min})$ = (9,154 – 0.001) / (155,324 − 0.001) = 0.059.

The *Exposure* for BTH is then:

$Exposure_{\mathrm{BTH}}$ = DF × *Magnitude*_BTH = 0.95 × 0.059 = 0.056,

Similarly, the $Exposure_{\mathrm{max}}$ and the $Exposure_{\mathrm{min}}$ among the 163 PMs are 0 and 0.345, respectively.

And the $Exposure_{normalized}$ of BTH is:

$Exposure_{\mathrm{normalized,BTH}} = (Exposure_{\mathrm{BTH}} - Exposure_{\mathrm{min}}) / (Exposure_{\mathrm{max}} - Exposure_{\mathrm{min}})$ = (0.056 − 0) / (0.345 − 0) = 0.162

Multiplying with the ToxPi score for BTH ($ToxPi_{\mathrm{BTH}}$ = 0.219) yields the risk index:

Risk index_BTH = $ToxPi_{\mathrm{BTH}} \times Exposure_{\mathrm{normalized,BTH}}$ = 0.219 × 0.162 = 0.0355.

The same procedure is applied to all other PMs.

Text S3 Literature review and compilation of PM occurrence data

To evaluate whether the detected PMs had been previously reported in water and to compile comparative concentration data, we carried out a targeted literature review in August 2025 and summarized the results in Table S5.

In a first step, we identified monitoring studies on persistent and mobile chemicals and related groups (micropollutants and emerging contaminants) in water by searching scientific publication databases (Google Scholar) using combinations of terms such as “persistent and mobile”, “PMT”, “vPvM”, “micropollutants”, “polar chemicals” or “emerging contaminants” with “water”, “groundwater”,

"surface water", "wastewater", or "drinking water". For each study, we screened the main text and, where available, the supplementary tables to extract the list of analyzed substances and their reported concentrations, matching them to our target list using CAS numbers, SMILES, or substance names. Where possible, reported concentrations (e.g., individual values, ranges, or summary statistics) were recorded for groundwater, surface water and wastewater and included in Table S5.

For compounds that could not be matched in this step, we performed additional searches using the substance name and known synonyms in combination with the same water-related keywords. If no occurrence data in any water matrix were found, the compound was classified as "first-time reported in water" in this study. If occurrence data were found only for wastewater, drinking water, surface water, or other water types such as rain and storm, but not for groundwater, the compound was classified as "first-time reported in groundwater".

Table S4. Criteria, attributes, method, and data sources used to calculate the ToxPi scores of the detected PM chemicals.

Criteria	Attributes	Unit	Scaling	Weight	Software
Persistence	Biowin1	unitless	-x	20/180	EPI Suite v4.1
	Biowin3	unitless	-x	20/180	
	Biowin5	unitless	-x	20/180	
Bioaccumulation	logBCF	unitless	x	30/180	EPI Suite v4.1
	logK_{ow}	unitless	x	30/180	
Toxicity	Carcinogenicity	unitless	x	6/180	VEGA v1.2.3
	Developmental toxicity	unitless	x	6/180	VEGA v1.2.3<br>T.E.S.T. v5.1.2.0<br>QSAR Toolbox v 4.6
	Mutagenicity	unitless	x	6/180	VEGA v1.2.3
	Endocrine disrupting effect	unitless	x	6/180	VEGA v1.2.3
	Hepatotoxicity	unitless	x	6/180	VEGA v1.2.3
	Skin irritation/corrosion	unitless	x	6/180	QSAR Toolbox v 4.6
	Eye irritation/corrosion	unitless	x	6/180	QSAR Toolbox v 4.6
	Skin sensitization	unitless	x	6/180	VEGA v1.2.3
	Repeated dose toxicity	mg/kg	$-\log(x) + \log(x)_{max}$	6/180	VEGA v1.2.3
	Oral rat LD50	mg/L	$-\log(x) + \log(x)_{max}$	6/180	T.E.S.T. v5.1.2.0
Mobility	logK_{oc}	unitless	$-x + (x)_{max}$	30/180	EPI Suite v4.1
	log*D* (pH=7.4)	unitless	$-x + (x)_{max}$	30/180	ChemAxon Chemicalize

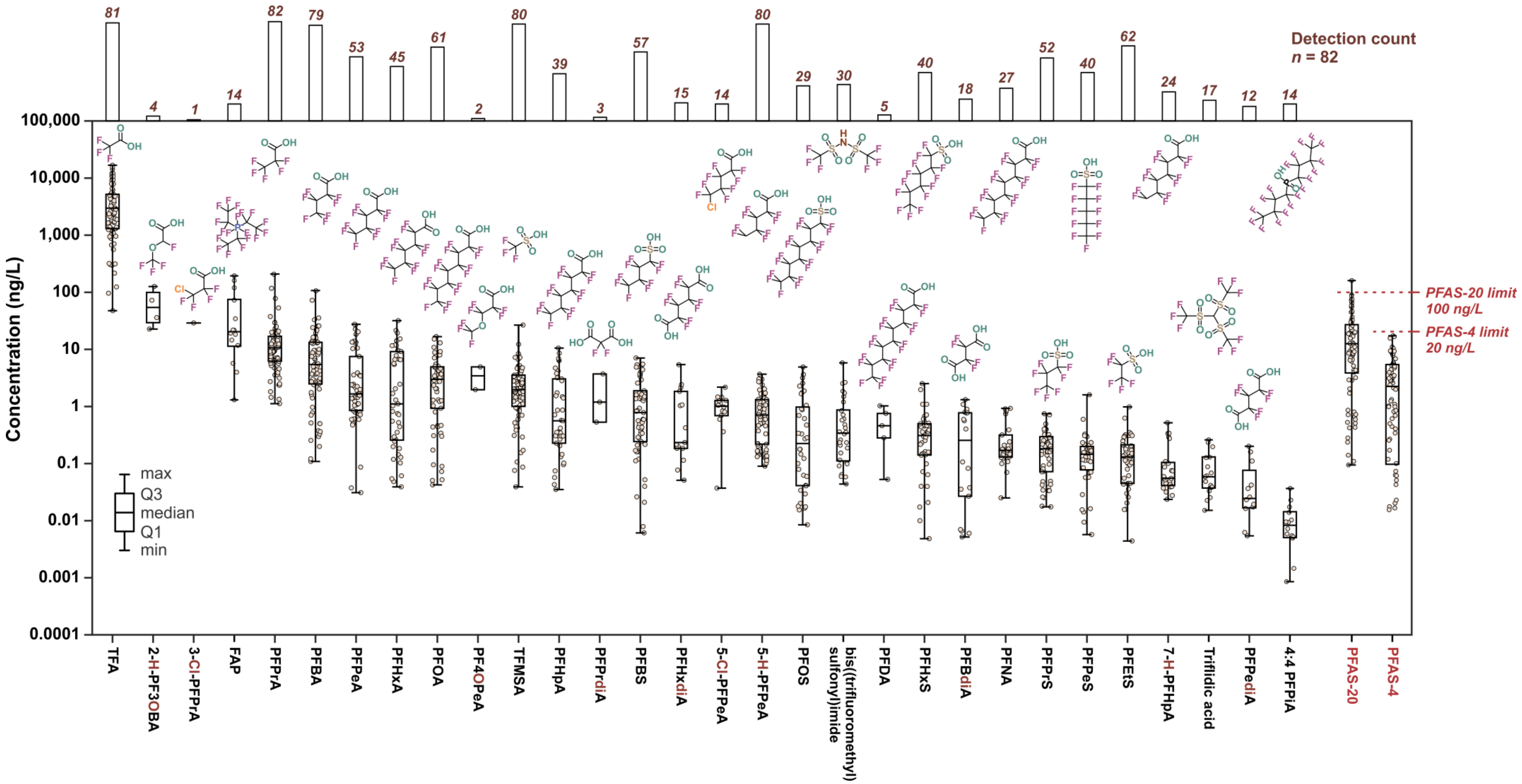

Fig. S1. Detection and concentrations of per- and polyfluoroalkyl substances (PFAS) in the 82 groundwater samples. The PFAS-20 comprises of the perfluoroalkyl carboxylic acids and perfluorosulfonic acids of C4 to C13, with a total limit of 100 ng/L as stipulated by Directive (EU) 2020/2184 on drinking water quality (https://eur-lex.europa.eu/eli/dir/2020/2184/oj), applicable from 2026 in Germany. The PFAS-4 consists of perfluoroctanoic acid (PFOA), perfluorononanoic acid (PFNA), perfluorohexanesulfonic acid (PFHxS), and perfluorooctanesulfonic acid (PFOS), with a total limit of 20 ng/L as set by Drinking Water Ordinance of 20 June 2023 (Federal Law Gazette 2023 I No. 159) (https://www.gesetze-im-internet.de/englisch_trinkwv/englisch_trinkwv.html#p0926), applicable from 2028 in Germany. See Table S6 for full data.

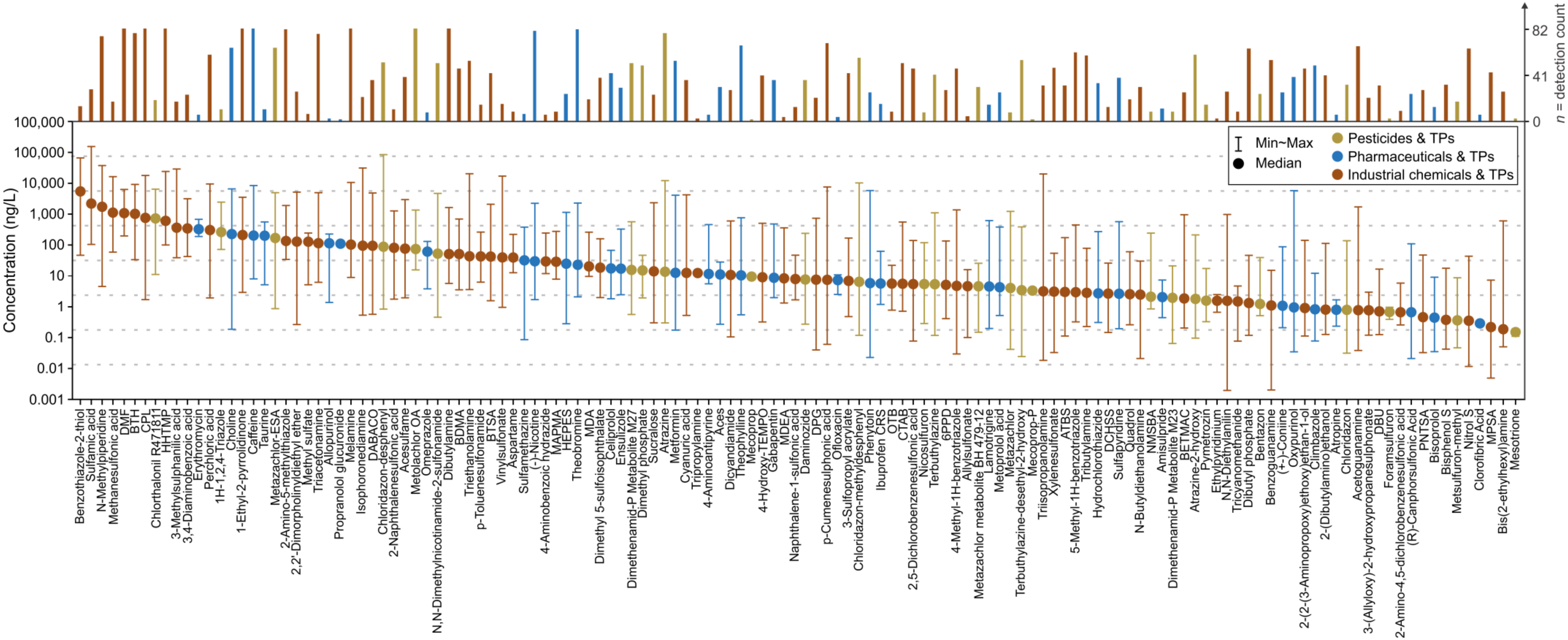

Fig. S2. Detection and concentrations of persistent and mobile chemicals apart from per- and polyfluoroalkyl substances which were presented in Fig. S1. See Table S6 for full data.

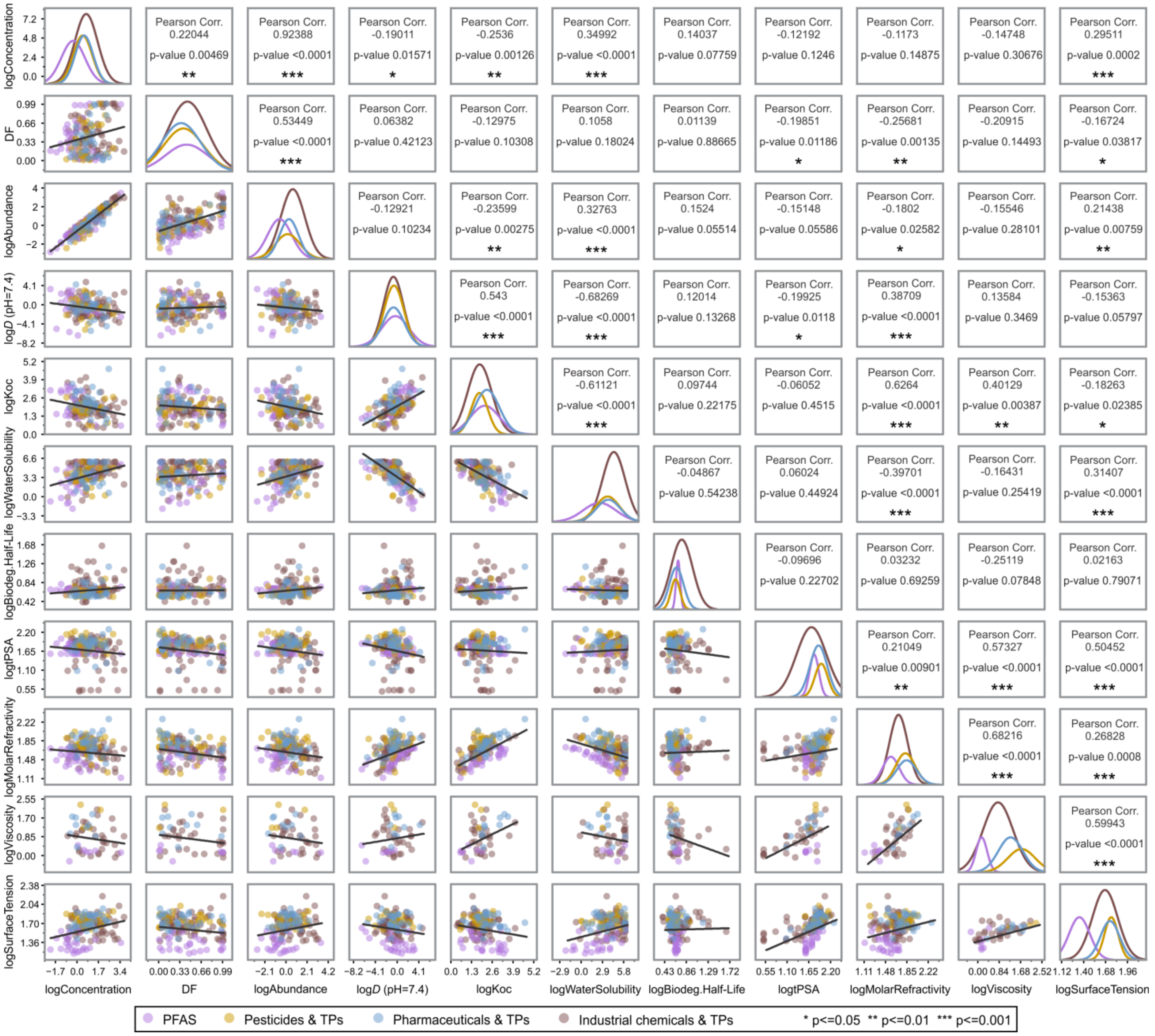

Fig. S3. Correlation analysis (Pearson, two-tailed test) between the distribution (median concentrations (ng L^{-1}), detection frequency (DF), and abundances) of individual persistent and mobile chemicals (PMs) and their physicochemical properties (n = 163). The abundance of each PM was calculated by multiplying the median concentration (n = detection count) by its DF. The log*D* (pH = 7.4) was predicted by ChemAxon Chemicalize (https://chemicalize.com/app/calculation), logK_{oc} values were obtained from EPI Suite v4.1 which does not include speciation correction based on environmental pH, water solubility data was sourced from ECOSAR v2.2, tPSA (topological polar surface area) values were calculated by ChemDraw 20.0, and all the other parameters were extracted from the ComTox Chemicals Dashboard v2.5.2 (https://comptox.epa.gov/dashboard/). The detailed source data is given in Table S8.

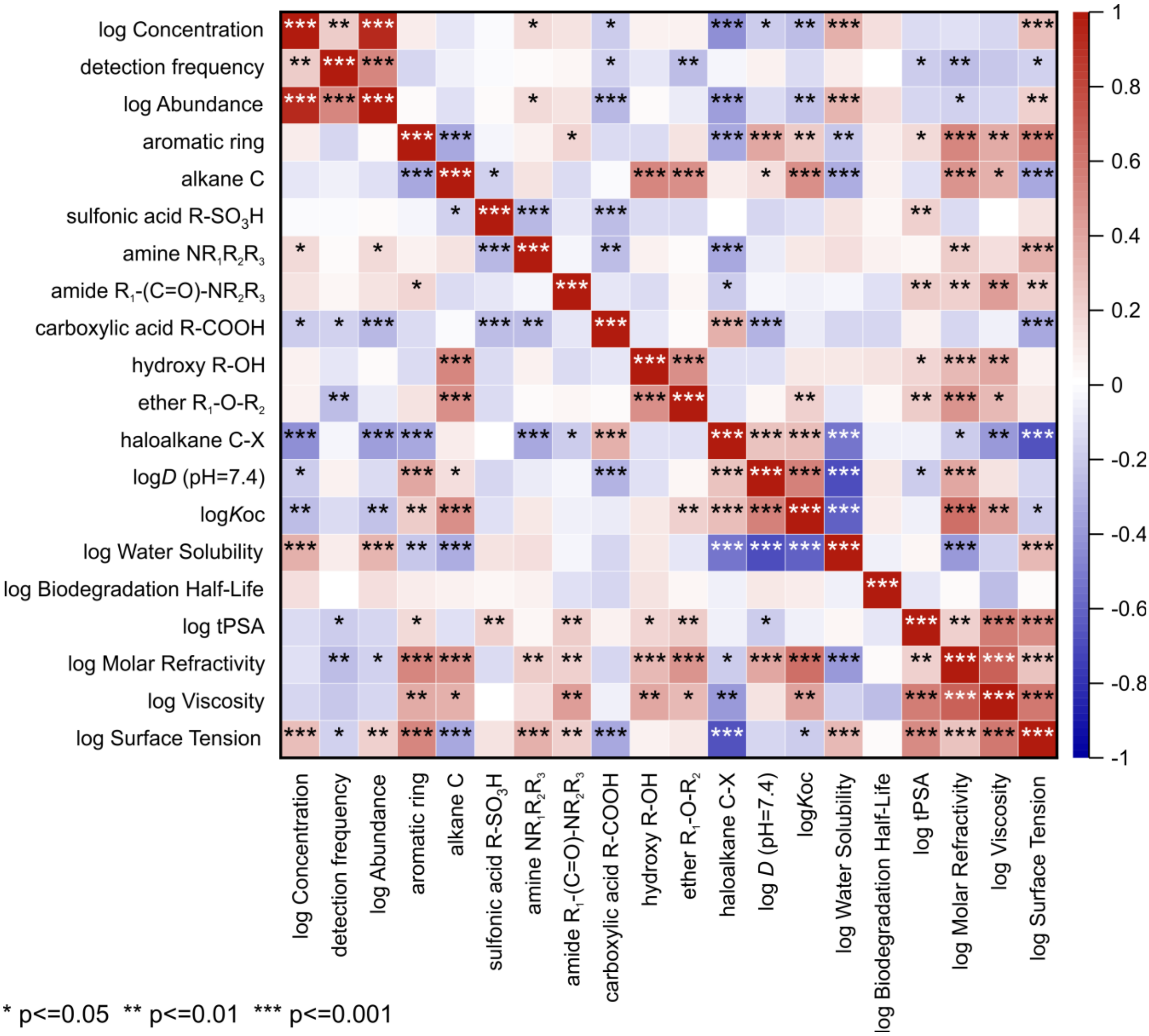

Fig. S4. Correlation analysis (Pearson) of persistent and mobile chemical (PM) functional groups with PM distribution (median concentrations (ng L^{-1}), detection frequency (DF), and abundances (calculated by multiplying the median concentration (n = detection count) by its DF)) and properties. The functional group data for PMs was represented as the count of each functional group within each chemical. To minimize bias, only functional groups present in more than 20 of the 163 detected PMs were included in the correlation analysis. The log*D* (pH = 7.4) was predicted by ChemAxon Chemicalize (https://chemicalize.com/app/calculation), logK_{oc} values were obtained from EPI Suite v4.1, water solubility data was sourced from ECOSAR v2.2, tPSA (topological polar surface area) values were calculated by ChemDraw 20.0, and all the other parameters were extracted from the ComTox Chemicals Dashboard v2.5.2 (https://comptox.epa.gov/dashboard/). The detailed source data is given in Table S8.

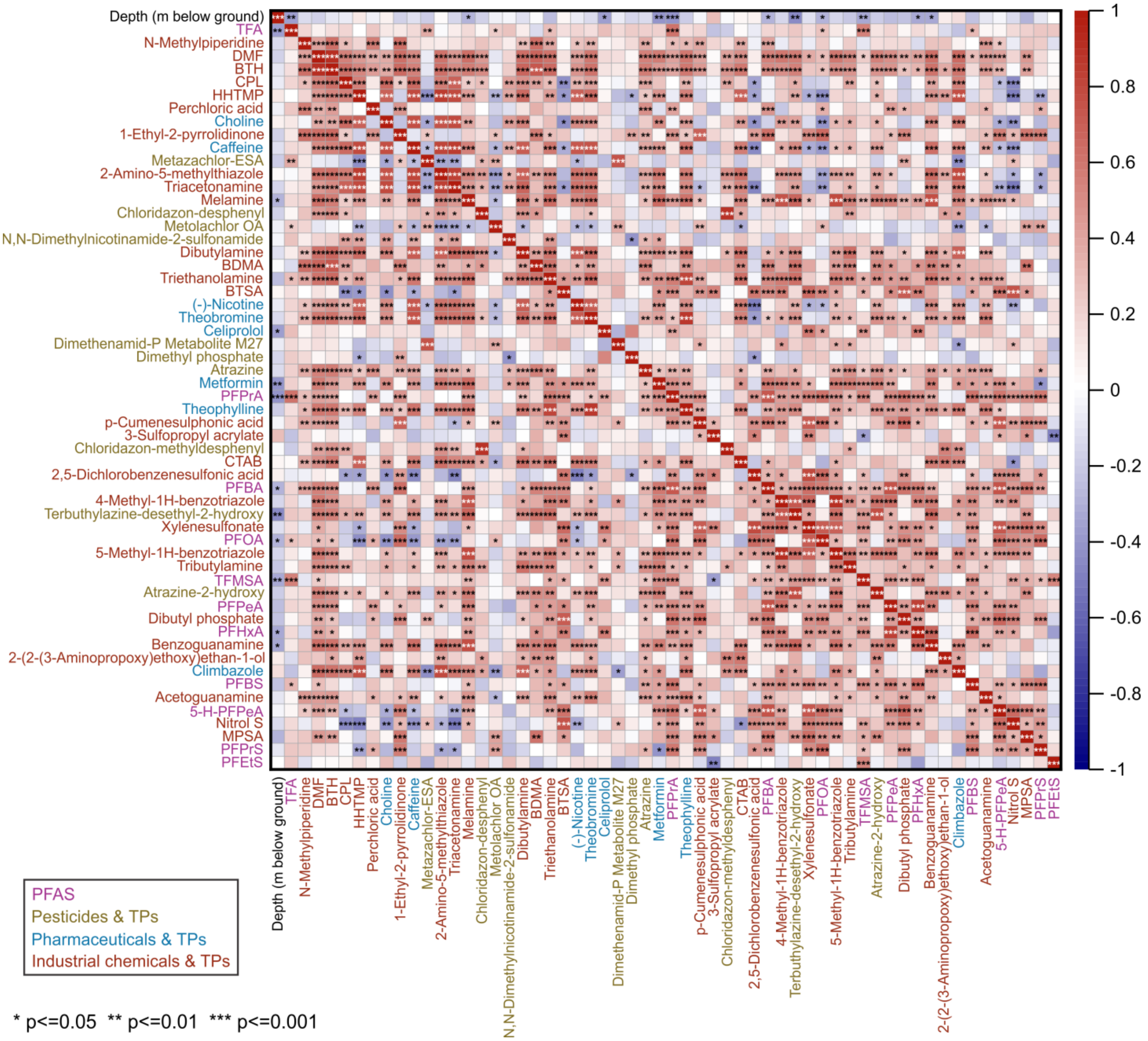

Fig. S5. Correlations (*n* = 82) between individual persistent and mobile chemical concentrations (detection frequency over 50%) and groundwater depths of the sampling sites (m below ground). All data used are log-transformed. Refer to Table S2 and Table S7 for detailed data.

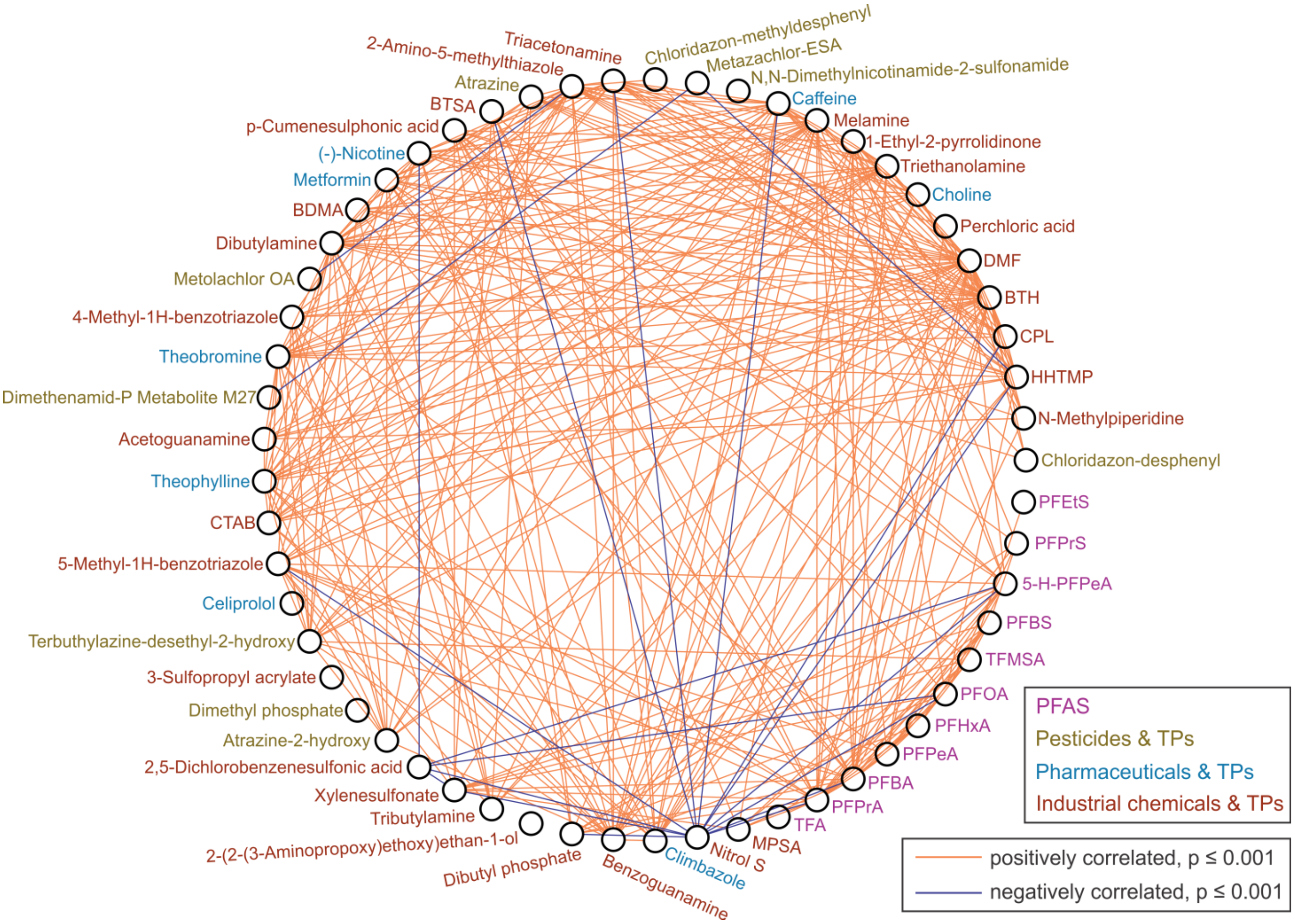

Fig. S6. Pearson correlation network plot displays very significant correlations ($p \leq 0.001$, shown with gray lines) among persistent and mobile chemicals (detection frequency over 50%). Specific *p*-values and Pearson's Corr. (r) between two significantly correlated (below 0.001) factors are presented in Table S9. All data used (n = 82) have been log-transformed. Refer to Table S7 for detailed data.

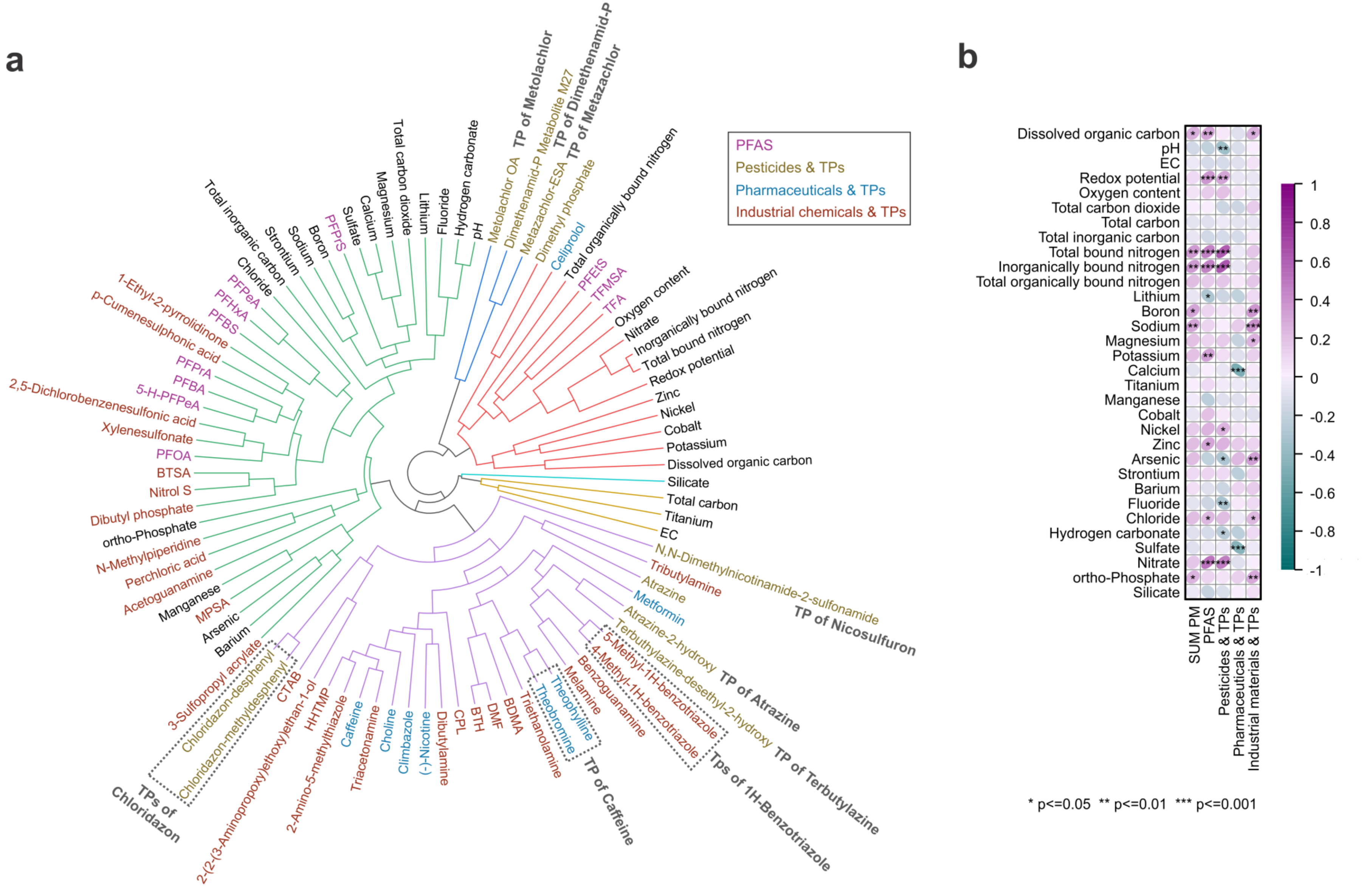

Fig. S7. Correlations between persistent and mobile chemical (PM) concentrations and groundwater characteristics (including property parameters and inorganic chemical concentrations). Panel a: Cluster analysis of groundwater characteristics and individual PM concentrations (detection frequency over 50%). Panel b: Pearson correlation analysis (*n* = 82) of grouped PM concentrations with groundwater characteristics. All data used, except pH values, are log-transformed. Refer to Table S3, Table S6, and Table S7 for detailed data.

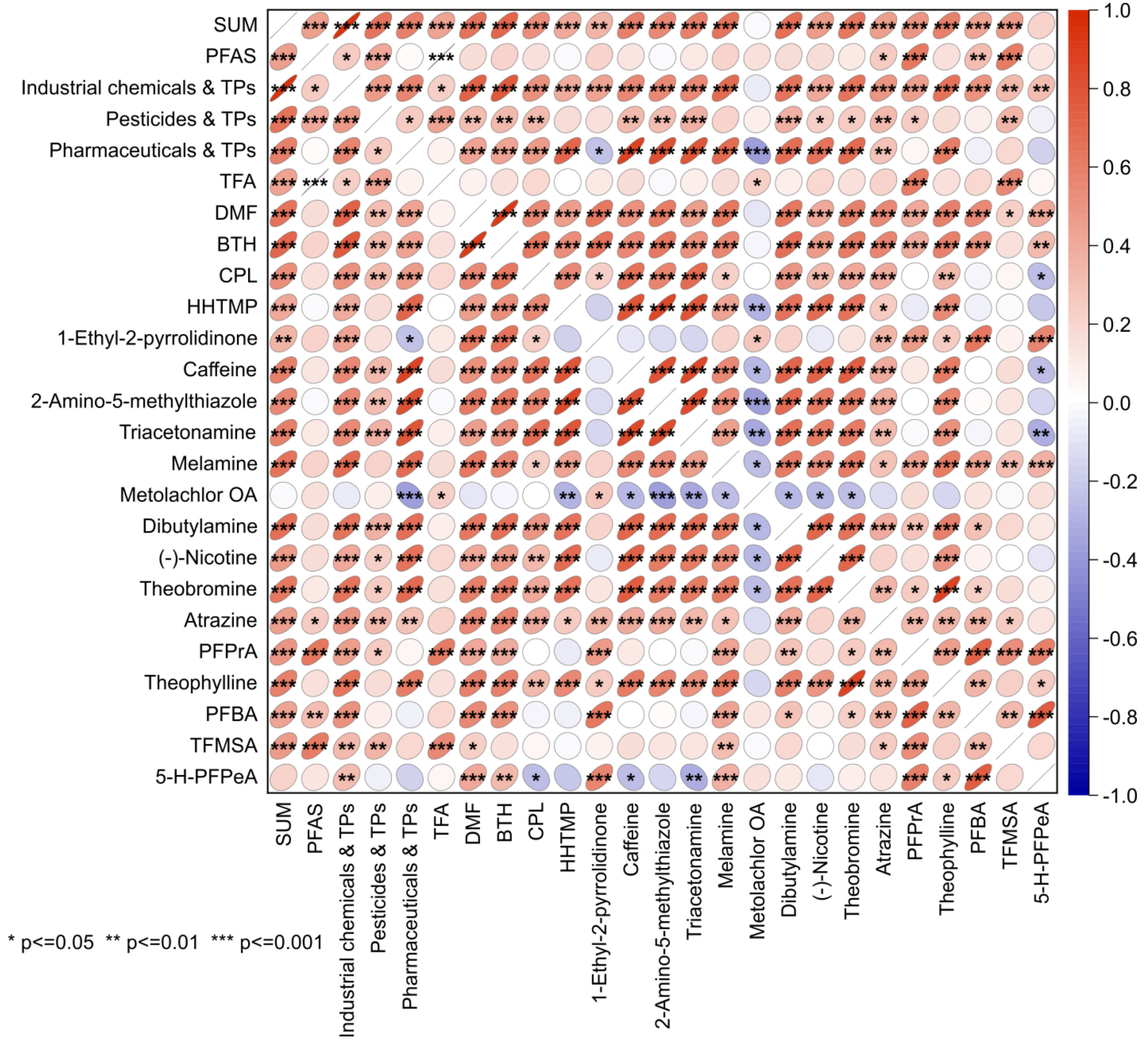

Fig. S8. Pearson correlation analysis (*n* = 82) of sum of all persistent and mobile chemicals and the four grouped chemical concentrations with the top ranked (of detection frequency) 20 individual chemical concentrations. All data used have been log-transformed. Refer to Table S6 and Table S7 for detailed data

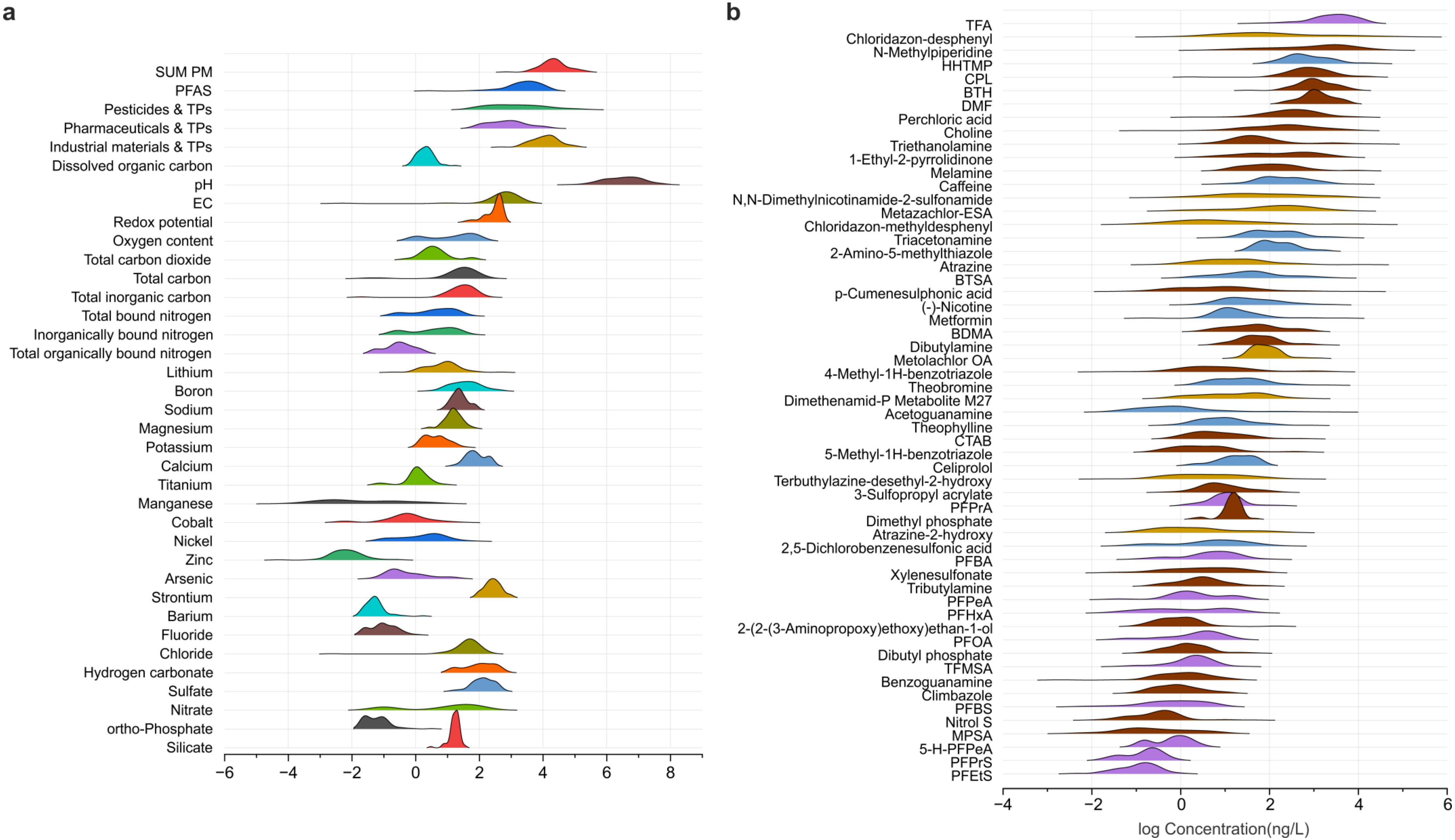

Fig. S9. Illustration of the value distributions of groundwater characteristics and grouped persistent and mobile chemical (PM) concentrations (Panel a, log-transformed data except for pH values, *n* = 82), along with individual PM concentrations detected in more than 50% of samples (Panel b; different colors indicate different chemical groups: purple means PFAS, yellow means pesticides and TPs, blue means pharmaceuticals and TPs, red means industrial chemicals and TPs). Specific data and the corresponding units are provided in Tables S3, Tables S4, and Tables S5.

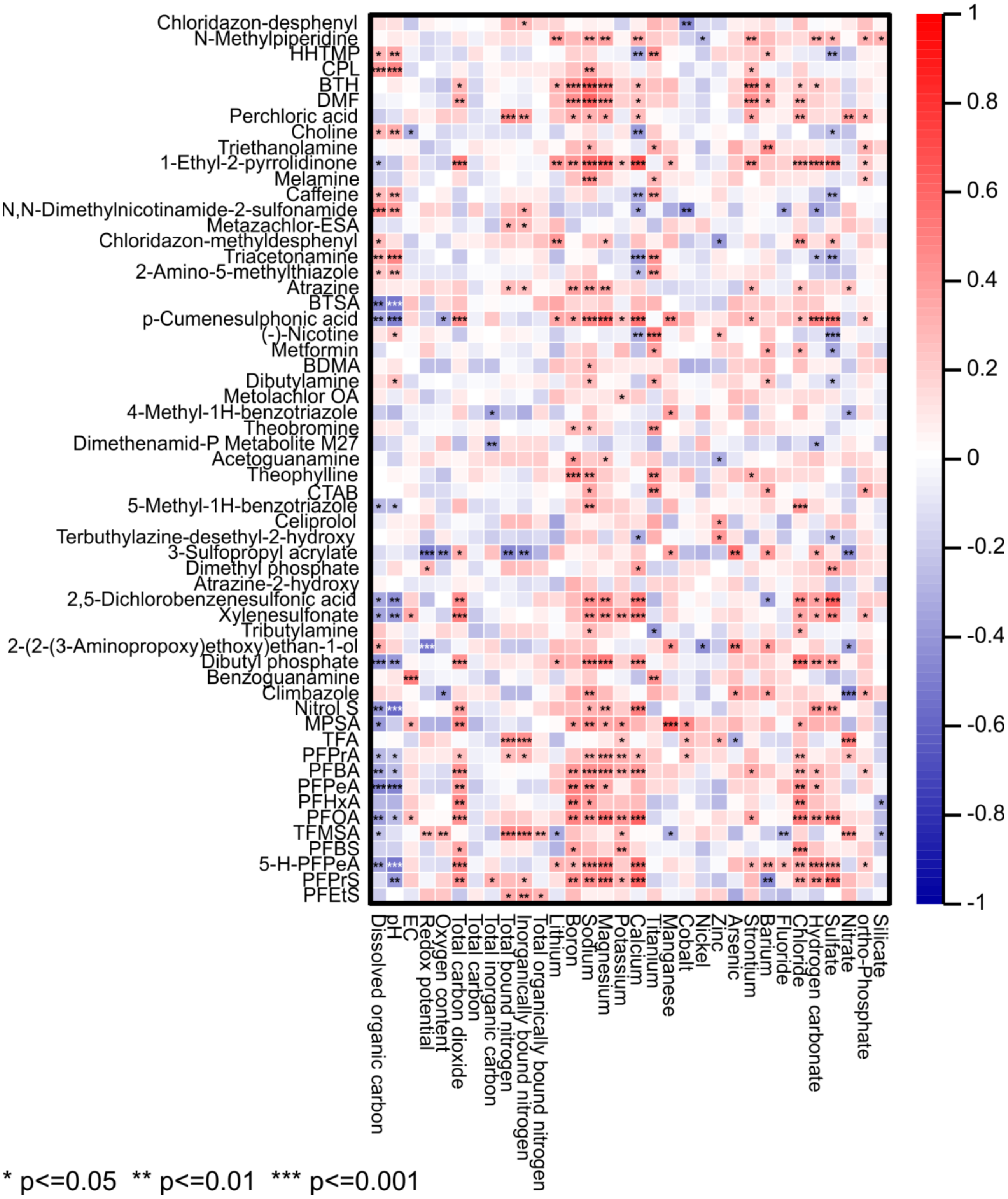

Fig. S10. Pearson correlation analysis (*n* = 82) of the values of groundwater characteristics with individual persistent and mobile chemical concentrations detected in over 50% of samples. All data used, except pH values, have been log-transformed. Refer to Table S3 and Table S7 for detailed data.

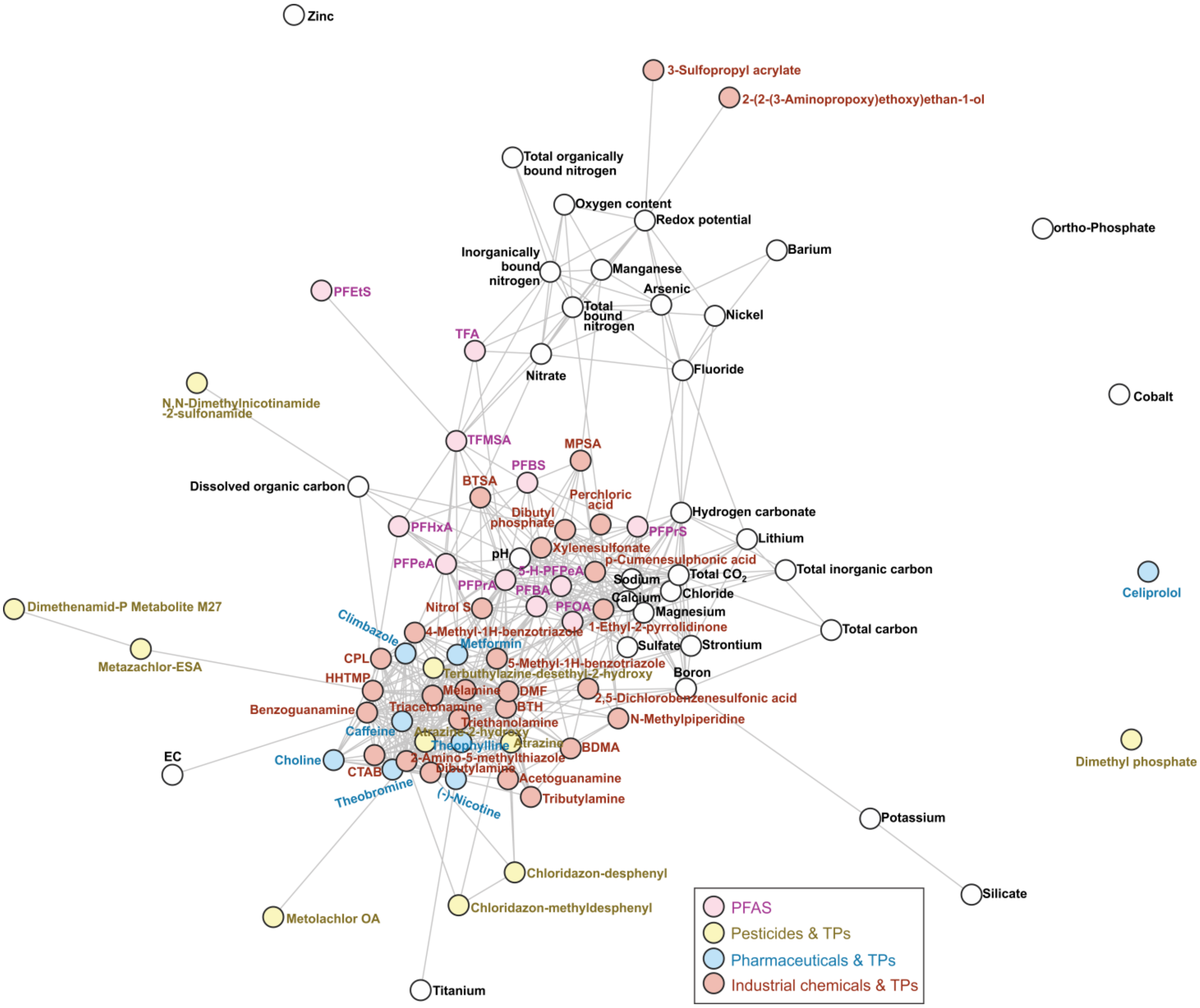

Fig. S11. Pearson correlation network plot displays very significant correlations ($p < 0.001$, shown with gray lines) among various groundwater characteristics and persistent and mobile chemicals (detection frequency over 50%). The short the grey line the more significant the correlation. Specific *p*-values and Pearson's Corr. (r) between two significantly correlated (below 0.001) factors are presented in Table S9. All data used (*n* = 82), except pH values, have been log-transformed. Refer to Table S3 and Table S7 for detailed data.

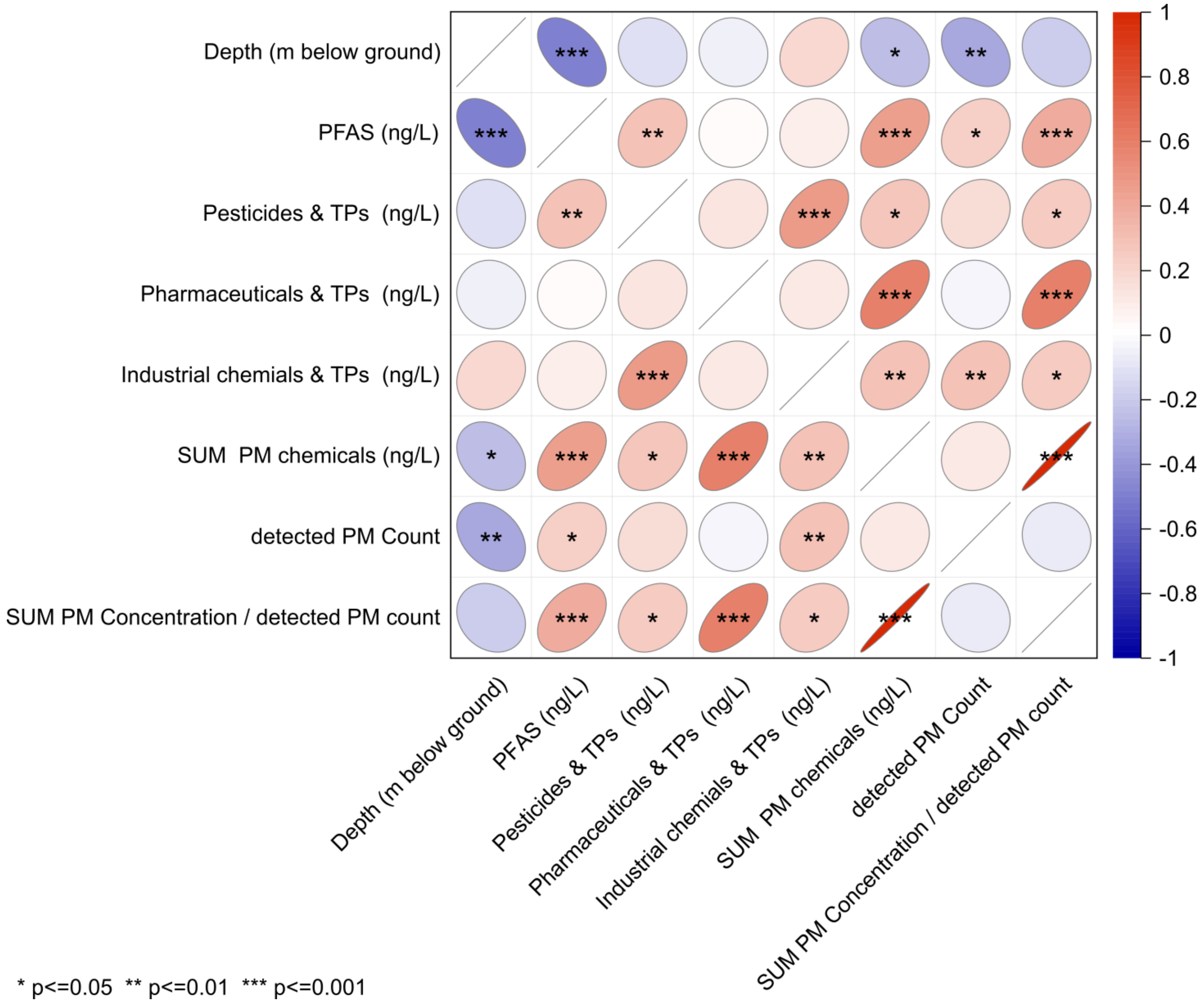

Fig. S12. Correlations (n = 82) between grouped and total persistent and mobile chemical (PM) concentrations (ng/L), the count of detected PMs, and groundwater depths (m below ground) at individual sampling sites. All data used are log-transformed. Refer to Table S2 and Table S6 for detailed data.

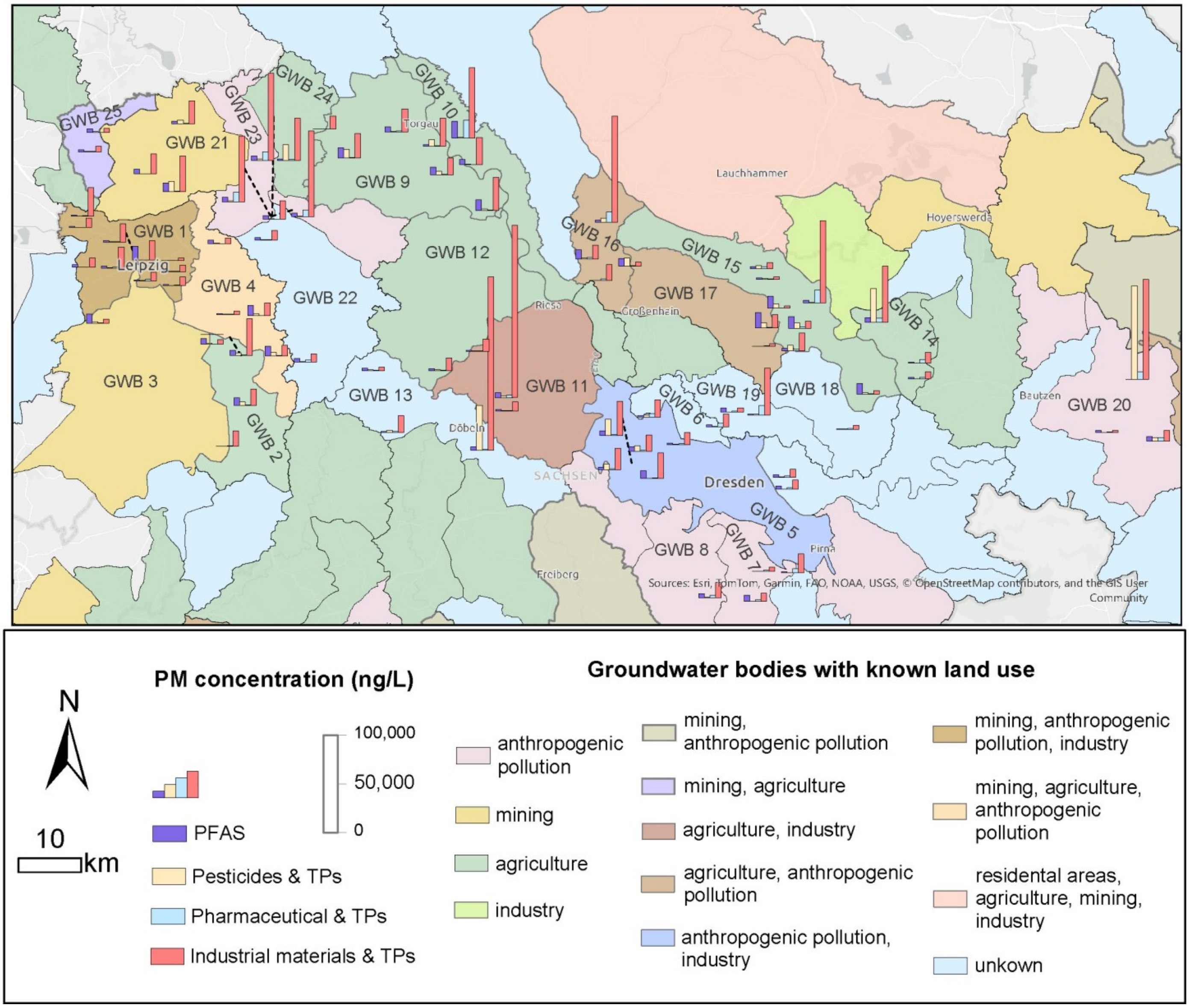

Fig. S13. The geographical distribution of the persistent and mobile chemicals (PMs) as four main groups, as a complement of Figure 3, across the observed groundwater bodies (GWB) with different known land use. See Table S6 for the detailed data. The data that presents the land use statues and the boundaries of the GWB across Saxony, Germany, was extracted from the iDA section of the website of Saxon State Ministry for Energy, Climate Protection, Environment and Agriculture (Sächsisches Staatsministerium für Energie, Klimaschutz, Umwelt und Landwirtschaft, https://www.umwelt.sachsen.de/umwelt/infosysteme/ida).

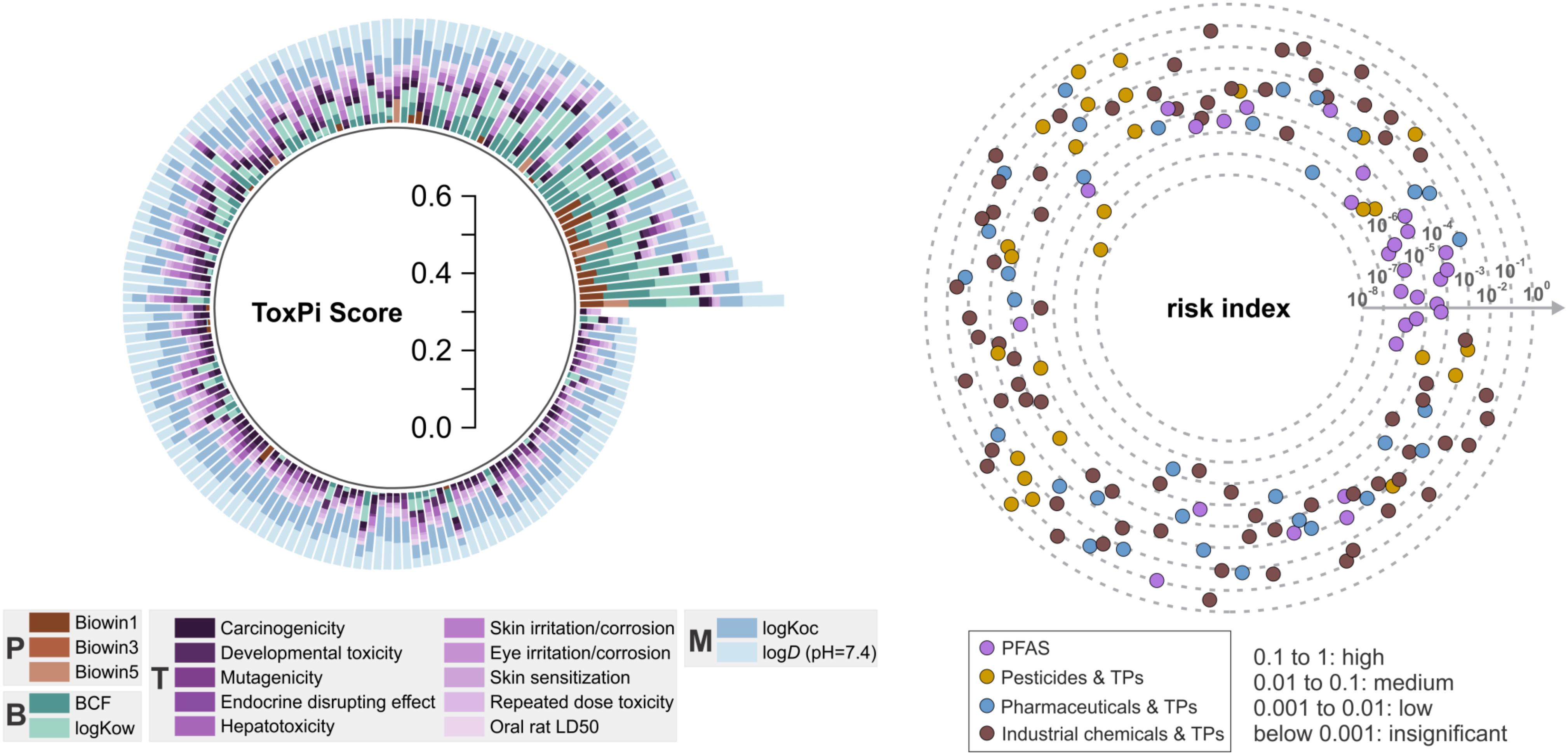

Fig. S14. The calculated ToxPi score (left panel) and risk index (right panel) of each persistent and mobile chemical. P: persistence, B: bioaccumulation, T: toxicity, M: mobility. The chemicals are displayed in anticlockwise order in both panels sorted according to their ToxPi ranking (as shown in Table S12).

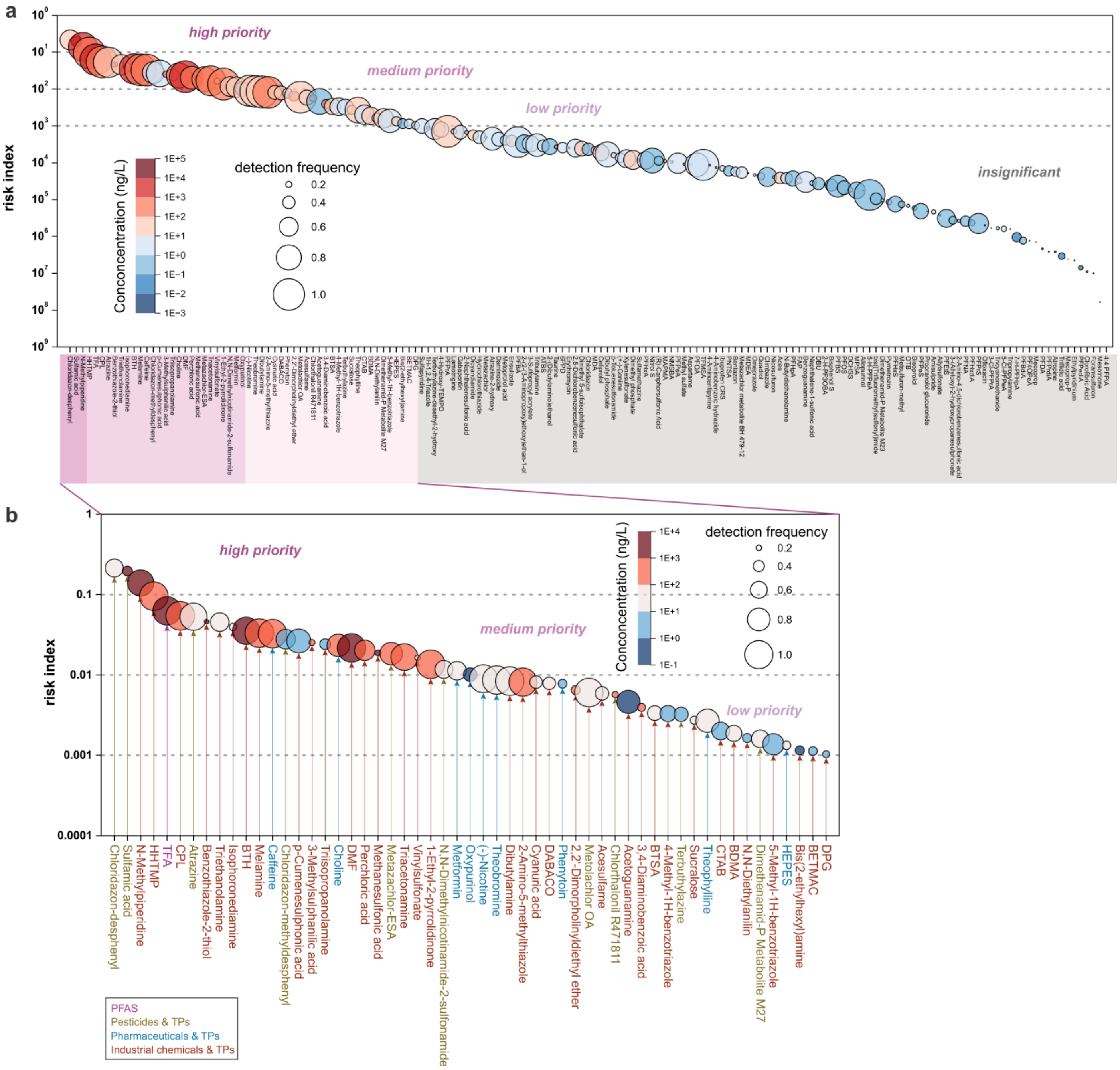

Fig. S15. Risk index of each persistent and mobile chemicals (PMs): (a) all the detected PMs; (b) chemicals whose risk index are higher than 0.001. The concentrations used correspond to the median values across all detected samples. See Table S12 for detailed data.

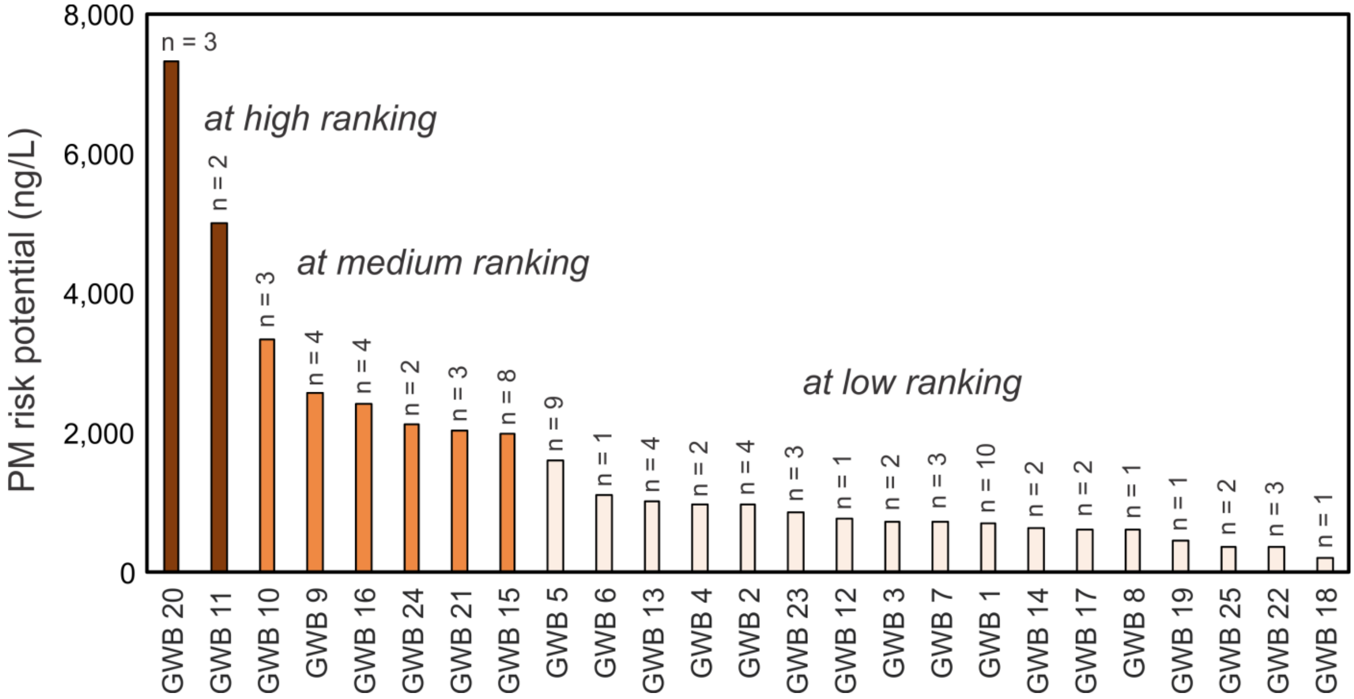

Fig. S16. Ranking of the groundwater bodies GWB based on their risk potentials according to the distribution of the 33 prioritized persistent and mobile chemicals (PMs) and their risk indices. The PM risk potential for each sampling site was calculated by multiplying each prioritized chemical's concentration (ng/L) by its respective risk index (unitless) and then summing the resulting values (∑ (Concentration × risk index)). The mean value across all sampling sites (n) within a given GWB was used to represent the risk potential for that GWB. Based on the resulting data, GWB with values above 4,000 ng/L are ranked highest, those between 2,000 and 4,000 ng/L are with medium ranking, and those below 2,000 ng/L are with low ranking.
